# Living Donor Liver Transplantation Versus Deceased Donor Liver Transplantation for Hepatocellular Carcinoma and HCV Patients: An Initial Umbrella Review

**DOI:** 10.3390/jcm14093047

**Published:** 2025-04-28

**Authors:** Ying Yang, Yu-Cheng He, Yun-Shi Cai, Ying-Hao Lv, Chang Liu, Hong Wu

**Affiliations:** 1 Department of General Surgery, West China Hospital, Sichuan University, Chengdu 610041, China; 2 Liver Transplant Center, Transplant Center, West China Hospital, Sichuan University, Chengdu 610041, China; 3 Division of Abdominal Tumor Multimodality Treatment, Cancer Center, West China Hospital, Sichuan University, No. 37 Guoxue Alley, Chengdu 610041, China

**Keywords:** living donor liver transplantation, deceased donor liver transplantation, hepatocellular carcinoma, meta-analysis, umbrella review

## Abstract

**Background**: Living donor liver transplantation (LDLT) has become a widely accepted alternative to deceased donor liver transplantation (DDLT). Nevertheless, the available meta-analyses shed light on a perplexing issue regarding which transplant is better. Therefore, we performed an umbrella review to summarize and evaluate the evidence from current meta-analyses. **Methods**: Two independent reviewers conducted a search of PubMed, Embase, Web of Science, and the Cochrane Database of Systematic Reviews from inception to 1 June 2024. The methodological quality of each included meta-analysis was evaluated using AMSTAR2 (A Measurement Tool to Assess Systematic Reviews). **Results**: The search identified 10 meta-analyses from 486 individual articles, including cohort studies and observational studies. Regrettably, the quality of these meta-analyses ranged from critically low to moderate. Receipt of LDLT offers a survival advantage to the patients with HCC compared with DDLT but with a higher complication rate. However, high-quality studies are required in the future to validate our assertions owing to the low certainty of the evidence. **Conclusions**: Despite the complication risks, LDLT remains a cost-effective option without compromising patient and graft survival, especially for HCC patients. Extensive, well-designed studies are essential to validate these conclusions.

## 1. Introduction

Globally, liver disease (cirrhosis, viral hepatitis, and liver cancer) causes over 2 million deaths annually, accounting for 4% of all deaths worldwide [1]. Since the first successful orthotopic liver transplantation (LT) in 1967 by Starzl et al. [2], LT has been the definitive treatment for end-stage liver disease and select liver malignancies. However, the demand for LT far exceeds supply. Recent data from the Organ Procurement and Transplantation Network (OPTN) and Scientific Registry of Transplant Recipients (SRTR) reveal 11,771 adult patients awaiting LT and the absolute number of those patients is greater than ever before [3]. The growing great discrepancy between the demand and supply of deceased donor livers has become the major impediment to the further expansion of LT, especially in Asia regions influenced by social customs and religious and cultural beliefs [4].

Since the first living donor liver transplantation (LDLT) was successfully performed by Strong et al. [5] in 1989, LDLT has become a pivotal strategy to address the global shortage of donor organs. While LDLT now serves as a viable alternative to deceased donor liver transplantation (DDLT), its technical complexity demands advanced surgical expertise and multidisciplinary coordination among surgeons, anesthesiologists, and intensive care teams. Early studies highlighted higher complication rates in LDLT, including biliary strictures, hepatic artery thrombosis and small-for-size syndrome, alongside inferior short-term survival outcomes compared to DDLT. However, refinements in surgical techniques (e.g., microvascular anastomosis, graft size optimization) and perioperative management have significantly improved LDLT safety and efficacy over the past decade. Consequently, LDLT accounts for >90% of liver transplants in Asia, where cultural and religious barriers limit deceased donor availability [4]. In contrast, LDLT adoption remains low in Western countries (5% of transplants), reflecting persistent concerns about donor risks and institutional experience.

Emerging evidence highlights the distinct benefits of living donor liver transplantation (LDLT), particularly in reducing mortality rates among patients on transplant waitlists and facilitating timely transplantation for critically ill individuals [6,7]. For patients with hepatocellular carcinoma (HCC) who meet the Milan criteria, LDLT offers comparable 5-year survival rates to DDLT while reducing the risk of tumor progression during extended waiting periods [8]. Pediatric recipients of LDLT also exhibit superior outcomes, with lower incidences of early graft dysfunction and postoperative infections [9,10]. However, there is ongoing debate regarding the potential association of LDLT with higher recurrence rates of HCC, which may be related to factors such as donor selection and surgical techniques [11].

To date, no randomized controlled trials (RCTs) have directly compared the outcomes of LDLT and DDLT. Existing meta-analyses of observational studies [6,8,12,13,14,15,16,17,18] constrained by heterogeneous outcome definitions, regional biases, and methodological inconsistencies, preclude definitive conclusions. To resolve this uncertainty, we conducted the first umbrella review synthesizing all available meta-analyses, with the aim of providing the most comprehensive assessment of LDLT and DDLT outcomes.

## 2. Materials and Methods

### 2.1. Umbrella Review Methods

This umbrella review was conducted in accordance with the guidelines for Preferred Reporting Items for Systematic Reviews and Meta-Analyses (PRISMA) [19] and the protocol was registered in PROSPERO (Registration ID: CRD42023477684). An umbrella review constitutes a secondary research methodology that systematically synthesizes and appraises existing systematic reviews or meta-analyses to comprehensively summarize high-level evidence in a research area. This approach is particularly advantageous in fields characterized by contentious topics, as it offers a more comprehensive and objective framework for understanding complex scientific inquiries.

### 2.2. Literature Search

We searched PubMed, Embase, Web of Science, and the Cochrane Database of Systematic Reviews from inception to 1 June 2024 for systematic reviews and meta-analyses of LDLT versus DDLT. The search strategy used the following terms/keywords: (living donor liver transplantation, LDLT, deceased donor liver transplantation, DDLT or cadaveric donor liver transplantation) AND (systematic review or meta-analysis), following the Scottish Intercollegiate Guidelines Network’s guidance [20]. Two authors independently screened the titles and abstracts and selected articles that met the inclusion criteria by full-text reading. Any discrepancy that could not be resolved by consensus between the two reviewers was resolved by a third author. The reference lists of all included meta-analyses were also manually screened for additional eligible studies.

### 2.3. Eligibility Criteria

The eligibility criteria were systematic reviews and meta-analyses of observational and randomized controlled trials studies measuring the clinical outcomes of LDLT versus DDLT. If more than one study was published to research the same clinical outcome, the most recent study with the largest sample size was included. This umbrella review was limited to studies of adults and those studies focusing primarily on pediatric and pregnant populations were excluded. Non-English studies and studies on animal or/and in vitro studies were excluded. Moreover, meta-analyses without a clear description of methods including search strategy, eligibility criteria, data extraction, quality assessment, statistical analysis, assessment of publication bias, and heterogeneity were also excluded.

### 2.4. Data Extraction

The following information was independently extracted from each eligible study by two reviewers: first author’s name, publication year, type of study subjects (only hepatocellular carcinoma(HCC) patients, mixed patients), clinical outcomes, number of included studies, number of LDLT and DDLT participants, study design (cross-sectional studies, case–control studies, cohort studies and randomized controlled trial), estimated summary effect (RR, OR, HR, WMD, SMD with 95% confidence intervals and the corresponding *p* values), meta-analytic model used (random and fixed), and any reported estimate of heterogeneity (I^2^ statistic and Cochran’s Q test *p* value), and publication bias assessment (*p* value of Egger’s test or funnel plot). If a subgroup analysis was conducted in meta-analyses, we also extracted any reported estimate of subgroup analysis in meta-analyses. Any discrepancies were resolved by consensus with a third reviewer.

### 2.5. Quality Assessment of Methods and Evidence

The methodological quality of eligible articles was evaluated by two independent reviewers using AMSTAR2 (A Measurement Tool to Assess Systematic Reviews) [21]. This evaluation system was composed of 16 items and classified the quality of evidence into four levels: high, moderate, low, or critically low.

### 2.6. Data Analysis

For each meta-analysis, the estimated summary effect size with 95%CI through fixed or random effects models was presented. Moreover, Cochran’s Q test and Egger’s test were extracted to evaluate the heterogeneity and publication bias, respectively, if they were available. The I^2^ metric was also used to assess the heterogeneity between studies [22]. Generally, the significance threshold was set to 0.05. While a *p* < 0.1 for Cochran’s Q test and Egger’s test were considered as statistically significant heterogeneity and publication bias, respectively. In addition, subgroup analyses were performed to further explore the potential sources of heterogeneity according to study design, sample size, Milan criteria, region of transplant, and so on. Forest plots were drawn using “forestploter” packages in R software (version 4.3.1).

## 3. Results

### 3.1. Characteristics of Included Meta-Analyses

**Figure 1:**
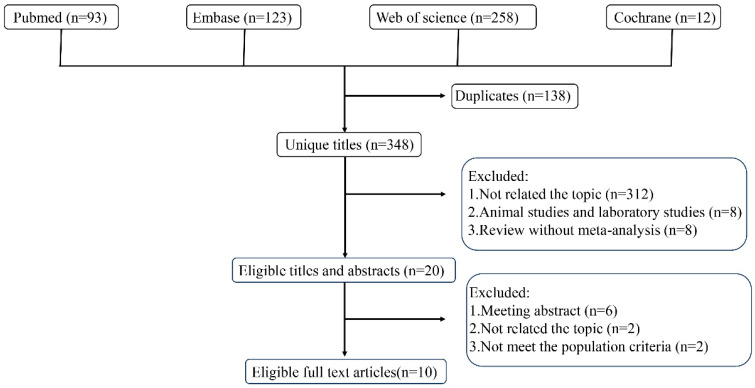
Flowchart of systematic search and selection process.

A total of 486 potential studies were identified by the systematic search of different databases (Figure 1). Finally, 10 meta-analyses [6,8,12,13,14,15,16,17,18,23] were included in this umbrella review and Table 1 summarizes the detailed characteristics of these studies.

### 3.2. The Survival of Recipient Patients

**Figure 2:**
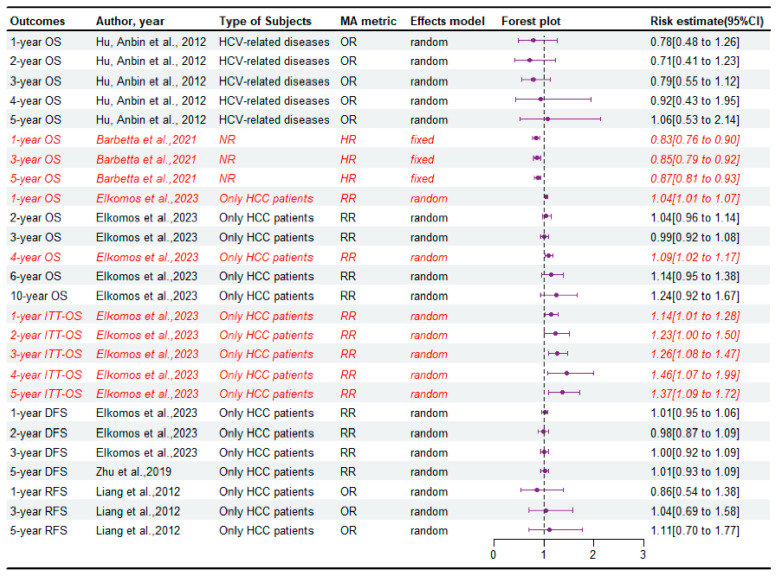
Forest plot: summary effect estimates of meta-analyses comparing LDLT versus DDLT for the survival of recipient patients [6,8,13,18,23]. The red/italic area indicates statistical significance. HR, hazard ratio; RR, risk ratio; NR, not reported; OR, odds ratio; MA, meta-analyses; OS, overall survival; ITT-OS, intention-to-treat overall survival; DFS, disease-free survival; RFS, recurrence-free survival.

A total of eight meta-analyses [6,8,12,13,14,15,18,23] reported data on overall survival (OS), intention-to-treat (ITT) OS (ITT-OS), disease-free survival (DFS), or recurrence-free survival (RFS) (Figure 2 and Supplementary Table S1). Of these, five [6,12,13,15,18] found a significant improvement in OS and ITT-OS in the LDLT group compared to DDLT, with Critically low quality of evidence according to the AMSTAR2 (Supplementary Table S1). Only one [14] article reported significant improvement in 3-year DFS in the DDLT group with Critically low quality of evidence according to the AMSTAR2 (Supplementary Table S1).

Subsequently, we summarized the subgroup analyses of these meta-analyses (Supplementary Table S2). For patients with HCV, subgroup analyses showed no significant differences in survival outcomes between the two groups. And these results of subgroup meta-analyses showed consistency with the primary findings. Similarly, subgroup analysis results of most meta-analyses showed no significant difference between the two groups in terms of survival outcomes including OS, DFS, and RFS. Notably, we did observe that the subgroup with larger sample sizes (greater than or equal to 300), especially those with larger LDLT samples (greater than or equal to 100) in the LDLT group tended to yield favorable OS compared with DDLT. In addition, a subgroup analysis conducted by Elkomos et al. [13] found that patients receiving LDLT in America had better 1-year OS than those receiving DDLT, while LDLT beyond the Milan criteria tended to achieve better 3-, 4-, 5-, and 10-year OS compared with DDLT. In contrast, a subgroup analysis in this study found that DDLT in America resulted in better 2- and 4-year DFS, and the implementation of DDLT within the Milan criteria achieved longer DFS.

### 3.3. The Disease Relapse

**Figure 3:**
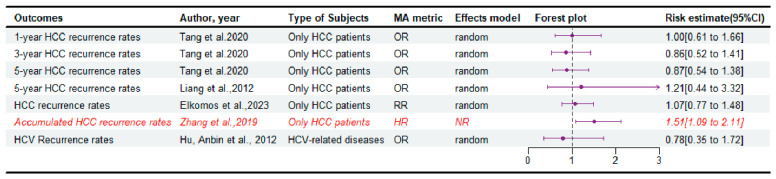
Forest plot: summary effect estimates of meta-analyses comparing LDLT versus DDLT for the disease relapse [8,13,15,17,23]. The red/italic area indicates statistical significance. HR, hazard ratio; RR, risk ratio; NR, not reported; OR, odds ratio; MA, meta-analyses.

Five meta-analyses [8,13,15,17,18] reported data on disease relapse, and only one paper found significant improvements in the DDLT group compared to LDLT, with critically low quality of evidence according to the AMSTAR2 (Figure 3 and Supplementary Table S3).

Consistent with the main results, subgroup analyses did not demonstrate significant differences in HCV recurrence rates between the two groups (Supplementary Table S4). Subgroup analyses revealed that the recurrence rates of HCC at 1, 3, and 5 years were largely consistent with the primary outcomes, with negligible differences observed among the groups. However, in the subgroup analysis [15] for sample size, the analysis showed that DDLT had significantly higher 1- and 5-year recurrence rates of HCC compared with LDLT when the sample size of DDLT is less than 100. Conversely, another subgroup analysis [8] for studies adhering to the Milan criteria indicated that LDLT had a higher 1-year recurrence rate than DDLT. And subgroups with larger sample sizes (greater than or equal to 300), especially those with predominant sample proportion of LDLT in the LDLT group had a higher 3-year recurrence rate than DDLT.

### 3.4. The Survival of Graft

**Figure 4:**
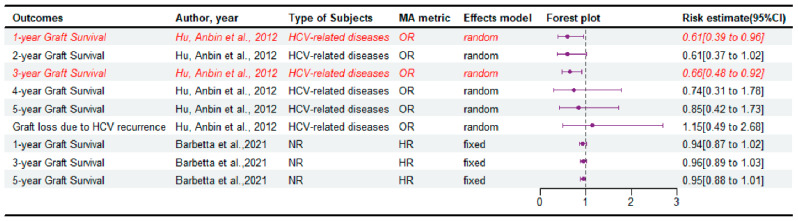
Forest plot: summary effect estimates of meta-analyses comparing LDLT versus DDLT for the survival of graft [6,23]. The red/italic area indicates statistical significance. HR, hazard ratio; NR, not reported; OR, odds ratio; MA, meta-analyses.

Only three meta-analyses [6,12,23] reported data on the graft survival (Figure 4 and Supplementary Table S5). Among them, Hu et al. [23] showed that DDLT significantly improved 1- and 3-year graft survival compared with LDLT in patients with HCV-related diseases. In contrast, Cavalcante et al. [12] showed that LDLT significantly improved 1-year graft survival compared with DDLT, despite the study population being unspecified (Supplementary Table S5). As shown in the Supplementary Table S6, most subgroup analyses showed no difference in graft survival between the two groups. A subgroup analysis [12] stratified by recipients’ age showed that LDLT was associated with superior one-year graft survival rates compared to DDLT. In contrast, DDLT has also been reported in association with increased graft survival in several subgroup analyses in another study [23]: 1-and 3-year graft survival (in the subgroup of prospective studies), 2- and 4-year graft survival (Ratio of LDLT/DDLT less than 0.5) and 1- year graft survival (in the subgroup of more presence of HCC patients in LDLT).

### 3.5. Perioperative Outcomes

**Figure 5:**
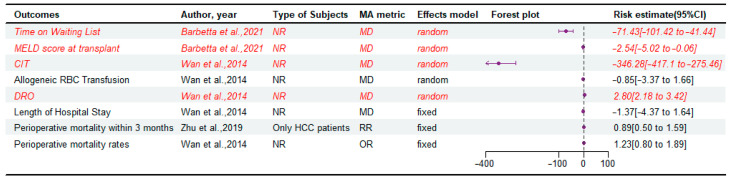
Forest plot: summary effect estimates of meta-analyses comparing LDLT versus DDLT for the perioperative outcomes [6,16,18]. The red/italic area indicates statistical significance. RR, risk ratio; NR, not reported; OR, odds ratio; MA, meta-analyses; MD, mean difference; DRO, duration of the recipient operation; RBC, red blood cell; CIT, cold ischemia time.

Although four meta-analyses [6,15,16,18] attempted to compare perioperative outcomes between LDLT and DDLT, only one meta-analysis [18] identified the study population (only HCC patients) (Figure 5). And in this study, HCC patients treated with LDLT are thought to have similar perioperative mortality rates within 3 months to those treated with DDLT. LDLT is associated with a shorter time on the waiting list and lower MELD scores at the time of surgery than DDLT [6]. LDLT typically requires more operative time (duration of the recipient operation, DRO) than DDLT, but LDLT has advantages in reducing cold ischemia time (CIT) and there is no significant difference in the need for allogeneic red blood cell (RBC) transfusion [15,16] (Supplementary Table S7). In addition, there was no significant difference between LDLT and DDLT in terms of length of hospital stay and postoperative mortality rates after transplantation [6,15,16,18] (Supplementary Table S7). As shown in Supplementary Table S8, subgroup analysis showed that LDLT had significantly shorter CIT compared with DDLT in both eastern and western regions. This conclusion was completely consistent with the subgroup analysis of sample size. And, consistent with the main results, subgroup analyses revealed that LDLT exhibited a longer operative duration compared to DDLT across both prospective and retrospective cohort studies, but there was no significant difference in allogeneic RBC transfusion requirement between the two groups.

### 3.6. Postoperative Complications and Retransplantation Rate

**Figure 6:**
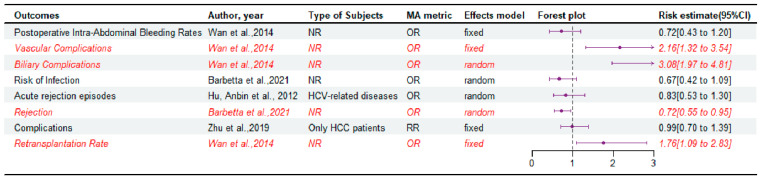
Forest plot: summary effect estimates of meta-analyses comparing LDLT versus DDLT for the postoperative complications and retransplantation rate [6,16,18,23]. The red/italic area indicates statistical significance. RR, risk ratio; NR, not reported; OR, odds ratio; MA, meta-analyses.

A meta-analysis of patients with HCC [18] showed that complication rates were comparable between LDLT and DDLT (Figure 6). For patients with HCV-related disease, the incidence of acute rejection was comparable between the LDLT and DDLT [23]. And the incidences of postoperative intra-abdominal bleeding rates and infection were also similar between LDLT and DDLT [6,16]. However, Barbetta et al. [6] showed that LDLT was associated with a lower incidence of rejection than DDLT when the study population was not limited to patients with HCC. However, most of the meta-analyses [6,15,16] reported that LDLT had a higher incidence of biliary complications, vascular complications, and retransplantation rates than DDLT (Supplementary Table S9). For HCV-related diseases, consistent with the main study results (Supplementary Table S9), subgroup analyses did not demonstrate significant differences in acute rejection between the two groups (Supplementary Table S10). Subgroup analyses based on the diagnosis of the patients showed that LDLT is associated with a higher rate of biliary complications, compared with DDLT in both the only HCC related and not HCC related groups. The subgroup analysis based on sample size also reached the same conclusion, indicating that LDLT is associated with a higher rate of biliary complications compared with DDLT under different sample sizes. And a subgroup analysis stratified by sample size indicated that when the sample size for LDLT exceeded 100, the incidence of vascular complications was significantly higher in LDLT compared to DDLT.

### 3.7. Heterogeneity

Two meta-analyses [14,17] presented low level heterogeneity (I^2^: 25~50%); while the clinical outcomes reported in eight meta-analyses [6,8,12,13,15,16,18,23] presented moderate-to-high level heterogeneity (I^2^: greater than or equal to 50%). And the subgroup analysis was conducted in these eight studies.

### 3.8. Publication Bias

Publication bias in meta-analyses was found for CIT, biliary complication rate, 1- and 3-year HCC recurrence rate (reported by Tang et al. [15]), and acute rejection episodes reported by Hu et al. [23].

### 3.9. Outcome of Quality Assessment

Table 2 shows AMSTAR2 item evaluations for included meta-analyses. Overall, of the ten papers, eight were of critically low quality, one was moderate quality, and one was low quality. In the critical domains, the most common methodological deficiencies are item 2 including four articles and item 13 including four articles, while in the non-critical domains, the most common methodological flaw is item 10 including ten articles. 

## 4. Discussion

LDLT has been a significant approach in LT for decades, aimed at addressing organ scarcity. However, its benefits and drawbacks have been inconsistently reported. This study presents the first umbrella review synthesizing multiple meta-analyses comparing LDLT with DDLT. Our findings indicate that LDLT is comparable to DDLT in recipient survival and offers advantages in perioperative outcomes, such as reduced waiting time and CIT. Despite this, LDLT faces challenges due to a higher incidence of postoperative complications, notably biliary complications. Additionally, the impact of LDLT on disease recurrence and graft survival remains inconclusive. This umbrella review presents the first comprehensive synthesis of meta-analyses, comparing clinical outcomes between LDLT and DDLT.

Our umbrella review suggested that LDLT might confer a survival advantage over DDLT for HCC patients. This advantage is likely due to superior graft quality and timely transplantation before disease progression. LDLT offers several theoretical benefits over DDLT [24], including reduced waiting times, shorter CIT, and more comprehensive donor screening, which collectively improve transplantation outcomes. The donor can be screened and evaluated more comprehensively before LDLT, ensuring optimal liver function and suitable physical conditions. Early studies [25,26] showed no significant difference in survival between LDLT and DDLT for HCC patients, with similar recurrence rates and OS. There have even been early reports of a worse prognosis with LDLT compared with DDLT [27]. However, subsequent studies [24,28,29,30,31,32,33,34] have corroborated its benefits in terms of OS, particularly in ITT survival rates, over DDLT. The A2ALL consortium [28] report underscores LDLT’s significant survival advantages, with a notably higher unadjusted 10-year survival rate compared to DDLT (70% vs. 64%) [35]. In 2019, two studies [29,32] confirmed that LDLT significantly reduced the risk of death and improved survival in HCC patients. A multicenter cohort study [31] involving 3052 patients, identified LDLT as an independent protective factor, reducing the overall risk of death by 49% (HR, 0.51; 95% CI, 0.36–0.71), highlighting substantial survival benefits of LDLT for HCC patients. Furthermore, a retrospective study [30] revealed that patients undergoing LDLT had better survival rates than those receiving DDLT. LDLT was also associated with a reduced need for intraoperative blood transfusions and post-transplant dialysis, as well as a significant 29.5% decrease in hospital costs. Similarly, in this umbrella review, the LDLT group exhibited significantly lower MELD scores compared to DDLT, aligning with the findings of the A2ALL cohort study [35]. In this study, only 16% of patients presented with a MELD score exceeding 20 in the LDLT group, while the corresponding proportion in the DDLT group was 43%. Our umbrella review found no significant differences in blood transfusion needs or postoperative hospital stays between the two groups, possibly due to the inclusion of older studies. Overall, LDLT offers improved OS for HCC patients, especially in ITT analyses, without increasing the necessity for blood transfusions or medical expenses.

In fact, the survival advantage of LDLT observed in our umbrella review and other studies is likely due to a lower dropout rate and reduced waiting times. This makes LDLT particularly beneficial in regions with a critical shortage of deceased donor organs, such as Asia [36], where it can provide a theoretically unlimited source of liver donors and prevent disease progression while waiting [32]. Differences in medical policies, ethics, and socioeconomic and cultural factors have made LDLT more prevalent in Asia, where over 90% of liver transplants use living donors, while the west relies more on DDLT. In the West, techniques like segmentation and extended donor use optimize DDLT [37]. This may lead to varying clinical outcomes for LDLT between regions. Subgroup analysis in our review also indicates that DDLT offers better survival outcomes in regions like the United States, where there is a larger pool of DDLT donors [37]. The impact of LDLT on postoperative disease progression in HCC patients is debated, with some studies [38,39] suggesting a higher recurrence rate with LDLT compared to DDLT. Proposed reasons include shorter wait-listing periods that may not allow sufficient tumor behavior assessment and the impact of liver regeneration after resection [37]. Additionally, the technical demands of LDLT, such as preserving the vena cava, hepatic artery, and bile duct [40], could potentially leave residual tumor or damage tumor capsules, influencing recurrence rates. Our umbrella review noted mixed results; while one study reported a higher cumulative HCC recurrence rate in LDLT group, some studies [26,41] found no significant difference in recurrence rates between two groups. The complexity of LDLT means better outcomes might occur where surgical expertise is high. A retrospective study [41] comparing 133 LDLT patients in Japan with 362 DDLT patients in the U.S. found similar 2-year recurrence rates, suggesting that surgical expertise (more surgical experience of LDLT in Japan) can mitigate differences in outcomes. Furthermore, differences in baseline characteristics, such as a higher proportion of salvage transplants in the LDLT group [38] and more bridging therapy (transarterial chemoembolization) in DDLT patients, could affect these results. For instance, the A2ALL study [42] found similar recurrence rates after adjusting for tumor and MELD prioritization. Notably, graft survival was comparable between LDLT and DDLT in our review.

For HCV-related liver disease, LDLT did not affect the survival outcomes of HCV patients and their graft compared with DDLT in this umbrella review, except for 1- and 3-year graft survival. Similarly, HCV recurrence rates were comparable between the two groups. Early findings by Shiffman et al. [43] demonstrated no significant differences in graft and patient survival, immunosuppression, acute rejection, and cytomegalovirus infection between LDLT and DDLT recipients over a 48-month follow-up. Additionally, liver inflammation and fibrosis scores were not significantly different between both groups within a 36-month timeframe. Similarly, another retrospective study [44] demonstrated comparable 2-year survival and graft survival rates, as well as histological recurrence rates between LDLT and DDLT. A multicenter retrospective study [45] also reported similar 3-year survival rates, although LDLT showed a lower 3-year graft survival rate, which normalized after excluding initial 20 cases. Risk factors for graft loss during the 90 days after LT included limited LDLT experience, pre-transplant HCC, and higher MELD scores at transplantation. The A2ALL cohort study [46], with a median follow-up of 4.7 years, found no significant differences in 5-year cumulative risk of advanced fibrosis, patient survival, and graft survival between LDLT and DDLT. A recent propensity score-matched cohort study [47] corroborated these findings, with no significant differences in 1-, 3-, and 5-year survival rates. Interestingly, a retrospective study [48] with an 8-year follow-up suggested superior outcomes with LDLT, as evidenced by higher overall survival and graft survival rates. Overall, this umbrella review and previous studies showed that LDLT did not compromise survival or graft outcomes in HCV-related liver disease patients, particularly when performed by experienced surgeons.

LDLT is a complex procedure requiring considerable expertise. Consequently, both our umbrella review and the previous literature indicated higher postoperative complication rates in LDLT recipients, especially biliary and vascular issues, compared to DDLT. The smaller size of bile ducts or vessels in LDLT necessitates anastomotic reconstruction, leading to higher biliary complications (7.4% to 39%), including bile leakage (5.1% to 23.4%) and bile duct strictures (6.5% to 21.5%) [49,50]. Vascular complications, such as bleeding and hepatic artery thrombosis, typically occur early post-LDLT (2.9% to 6%) [50,51,52]. Rejection rates varied, with some studies showing no difference between LDLT and DDLT [36], while others suggested a lower incidence in LDLT [6,53,54]. Infection risk was not significantly different between the two groups in our review, consistent with previous meta-analyses [6]. Undoubtedly, complications in LDLT were associated with a higher retransplantation rate in this umbrella review and previous reports (15.9% vs. 9.3%) [24]. Nevertheless, as surgical instruments and experiences improve, the complication rate is expected to decrease, especially after overcoming the learning curve [24,37]. For instance, microsurgery advancements have reduced biliary complications in LDLT within the Kaohsiung team [55], and combined microsurgical techniques with postoperative Doppler monitoring have nearly eliminated arterial thrombosis [56].

Another fact is that the prevalence of low-quality meta-analyses (8/10 studies rated as critically low by AMSTAR2) creates uncertainty in our conclusions. For instance, three meta-analyses [12,15,18] claiming better survival for LDLT lacked sensitivity analyses to test their findings’ robustness amid high heterogeneity (I^2^ > 75%). This oversight could lead to overestimating LDLT benefits, as unaccounted factors like regional surgical expertise and donor–recipient matching might skew results in low-quality studies. Additionally, the lack of sensitivity analyses limits our understanding of heterogeneity sources, such as study design or geographic differences. For instance, Asian studies often compared LDLT with “extended criteria” DDLT grafts, while Western studies focused on standard DDLT [4,57] potentially exaggerating LDLT benefits in Asian groups. This highlights the need for standardized subgroup reporting in future reviews.

The review acknowledges several limitations, including reliance on the meta-analyses of observational studies without randomized controlled trials, resulting in low-quality evidence. Confounding factors like surgical expertise and regional practices may affect outcomes, and not all studies conducted subgroup analyses. The focus on published meta-analyses might exclude unpublished studies, potentially skewing conclusions. Despite these issues, the review offers valuable insights into LDLT and DDLT outcomes. It highlights that LDLT provides similar long-term survival to DDLT in HCC/HCV patients and reduces waitlist mortality, crucial for areas with organ shortages. This supports expanding LDLT in Asia and the reconsideration of its limited use in Western regions due to safety concerns. Furthermore, our identification of increased biliary complications associated with LDLT emphasizes the necessity for standardized postoperative monitoring protocols, particularly in low-volume centers. We suggest three research priorities to address current limitations: (1) Conduct multicenter RCTs comparing LDLT and DDLT in matched HCC/HCV cohorts, with stratified randomization by MELD score and tumor stage. (2) Establish global prospective registries with large cohorts to collect detailed data on surgical techniques, immunosuppression, and donor–recipient matching. (3) Perform cost-effectiveness analyses of LDLT versus DDLT across various healthcare systems, considering both direct costs and indirect benefits.

## 5. Conclusions

LDLT is a promising alternative to DDLT, offering similar long-term outcomes and potentially lowering mortality for HCC patients by reducing wait times. However, it carries higher risks of biliary and vascular complications due to its complexity. These issues can be mitigated with increased surgical experience. Large-scale, high-quality studies are needed to confirm these findings.

## Figures and Tables

**Table 1:** Main Characteristics of the Included Articles.

Author/Year	Type of Databases (No. of Databases); Last Search	No. of Included Studies	Type of Subjects	Clinical Outcomes	Main Conclusion	AMSTAR2 Final Rating
Grant et al., 2013 [14]	MEDLINE, Embase, PubMed (3); April 2012	12	Only HCC patients	DFS, OS	Decreased DFS after LDLT compared with DDLT for HCC.	Critically low
Zhu et al., 2019 [18]	PubMed, Embase, Cochrane Library, Google Scholar, WanFang (5); January 2018	29	Only HCC patients	OS, ITT-OS, Recurrence rate, DFS, Perioperative mortality within 3 months, Complications	LDLT was not inferior to DDLT in consideration of comparable OS, DFS, recurrence rate, mortality within 3 months and postoperative complication rate, but a possible improvement in long-term intention-to-treat survival.	Critically low
Cavalcante et al., 2022 [12]	PubMed, Medline (2); June 2021	28	NR	OS, Graft survival	Better patient survival at 1, 3, and 5 years among patients who received LDLT, compared to DDLT, as well as better 1-year graft survival.	Critically low
Tang et al., 2020 [15]	PubMed, Embase, Cochrane Library (3); November 2019	39	NR	OS, HCC recurrence rate, CIT, RBC transfusion, DRO, Postoperative Intra-Abdominal Bleeding Rate, Perioperative Mortality, Length of Hospital Stay, Vascular Complication Rate, Biliary Complication Rate, Retransplantation Rate, HCV Recurrence Rate	LDLT was not inferior to DDLT in consideration of RBC transfusion, length of hospital stay, perioperative mortality, retransplantation rate, HCV recurrence rate, and HCC recurrence rate, but it was an improvement in CIT, postoperative intra-abdominal bleeding rate, and OS.	Critically low
Liang et al., 2012 [8]	PubMed, MEDLINE, Embase, Cochrane Library (4); NR	7	Only HCC patients	OS, RFS, tumor recurrence rates	Patient survival, recurrence, and RFS rates are at least comparable in HCC patients undergoing LDLT and HCC patients undergoing DDLT (especially in those meeting the Milan criteria).	Critically low
Zhang et al., 2019 [17]	Cochrane Library, PubMed, Embase (3); October 2017	7	Only HCC patients	Accumulated HCC recurrence rates	There is an overall increased risk for HCC recurrence in LDLT as compared with that of DDLT.	Critically low
Barbetta et al., 2021 [6]	PubMed, Embase and Embase Classic, Cochrane Library, Web of Science, Clinicaltrials.gov, Google Scholar (6); March 2018	19	NR	OS, Graft Survival, MELD score at transplant, Time on Waiting List, Hepatic artery thrombosis, Biliary complications, Risk of infection, Length of stay, Rejection	LDLT is associated with improved patient survival, less waiting time, and lower MELD at LT, despite posing a higher risk of biliary complications that did not affect survival posttransplant.	Critically low
Wan et al., 2014 [16]	MEDLINE, Embase, Cochrane Library (3); October 2013	19	NR	DRO, Allogeneic RBC Transfusion, Length of the hospital stay, CIT, Biliary complications, Vascular complications, intra-abdominal bleeding rates, perioperative mortality rates, retransplantation rates	LDLT is associated with a higher rate of surgical complications after transplantation.	Low
Elkomos et al., 2023 [13]	PubMed, Scopus, Web of Science, Cochrane Library (4); July 2021	35	Only HCC patients	OS, DFS, ITT-OS, Recurrence rates	LDLT provides much better survival benefits to HCC patients, especially in regions that suffer from low deceased organ availability.	Critically low
Hu, Anbin et al., 2012 [23]	PubMed, MEDLINE, EMBASE, Cochrane Library (4); NR	14	HCV-related diseases	OS, Graft Survival, Acute rejection episodes, HCV Recurrence, Graft loss due to HCV recurrence	LDLT was equivalent to DDLT in terms of patient survival, long-term graft survival, HCV recurrence, and acute rejection rates, with potentially lower short-term patient and graft survival.	Moderate

CIT: cold ischemia time, DDLT: deceased donor liver transplantation, DFS: disease-free survival, DRO: duration of the recipient operation, HCC: Hepatocellular carcinoma, HCV: hepatitis C virus, ITT-OS: intention-to-treat overall survival, LDLT: living donor liver transplantation, LT: liver transplantation, NR: not report, OS: overall survival, RBC: red blood cell.

**Table 2:** Quality assessment of included meta-analyses with AMSTAR 2.

Included Studies	ITEMS																Final Rating
	1	2	3	4	5	6	7	8	9	10	11	12	13	14	15	16	
Grant et al., 2013 [14]	Y	pY	N	pY	Y	Y	N	Y	pY	N	Y	N	Y	N	Y	N	Critically low
Zhu et al., 2019 [18]	Y	Y	N	pY	Y	Y	Y	Y	pY	N	Y	Y	N	Y	Y	Y	Critically low
Cavalcante et al., 2022 [12]	N	N	N	pY	Y	N	pY	N	pY	N	Y	Y	N	N	Y	N	Critically low
Tang et al., 2020 [15]	N	N	N	pY	Y	Y	Y	Y	pY	N	Y	N	N	Y	N	Y	Critically low
Liang et al., 2012 [8]	Y	N	N	pY	Y	Y	Y	Y	Y	N	Y	Y	Y	Y	Y	Y	Critically low
Zhang et al., 2019 [17]	Y	N	N	pY	Y	N	Y	Y	Y	N	Y	Y	Y	Y	N	Y	Critically low
Barbetta et al., 2021 [6]	Y	pY	N	Y	Y	Y	Y	Y	pY	N	Y	N	Y	Y	N	Y	Critically low
Wan et al., 2014 [16]	Y	pY	N	pY	Y	N	Y	Y	Y	N	Y	Y	Y	Y	Y	Y	Low
Elkomos et al., 2023 [13]	Y	pY	Y	pY	Y	Y	Y	Y	pY	N	Y	N	N	Y	Y	Y	Critically low
Hu, Anbin et al., 2012 [23]	Y	pY	Y	Y	Y	Y	Y	Y	Y	N	Y	Y	Y	Y	Y	Y	Moderate

The specific assessment methods for AMSTAR 2 were performed with reference to the literature reported by Shea et al. [21]. Abbreviations: Y, yes; pY, partial yes; N, no. AMSTAR 2 critical domains: PICO description (item 1); protocol registered before the commencement of the review (item 2); adequacy of the literature search (item 4); justification for excluding individual studies (item 7); risk of bias from individual studies being included in the review (item 9); source of funding of primary studies (item 10); appropriateness of meta-analytical methods (item 11); consideration of risk of bias when interpreting the results of the review (item 13); assessment of presence and likely impact of publication bias (item 15). Rating overall confidence in the results of the review: High: No or one non-critical weakness: the systematic review provides an accurate and comprehensive summary of the results of the available studies that address the question of interest. Moderate: More than one non-critical weakness: the systematic review has more than one weakness but no critical flaws. It may provide an accurate summary of the results of the available studies that were included in the review. Low: One critical flaw with or without non-critical weaknesses: the review has a critical flaw and may not provide an accurate and comprehensive summary of the available studies that address the question of interest. Critically low: More than one critical flaw with or without non-critical weaknesses: the review has more than one critical flaw and should not be relied on to provide an accurate and comprehensive summary of the available studies.

## Data Availability

The original contributions presented in this study are included in the article/Supplementary Materials. Further inquiries can be directed to the corresponding author(s).

## Supplementary Materials

The following supporting information can be downloaded at: https://www.mdpi.com/article/10.3390/jcm14093047/s1, Supplementary Table S1. Characteristics and quality assessment of the meta-analyses comparing survival outcomes between LDLT and DDLT in recipient patients. Supplementary Table S2. Available results of the subgroup meta-analyses comparing survival outcomes between LDLT and DDLT in recipient patients. Supplementary Table S3. Characteristics and quality assessment of the meta-analyses comparing the disease relapse between LDLT and DDLT in recipient patients. Supplementary Table S4. Available results of the subgroup meta-analyses comparing the disease relapse between LDLT and DDLT in recipient patients. Supplementary Table S5. Characteristics and quality assessment of the meta-analyses comparing the survival of graft between LDLT and DDLT. Supplementary Table S6. Available results of the subgroup meta-analyses comparing the survival of graft between LDLT and DDLT in recipient patients. Supplementary Table S7. Characteristics and quality assessment of the meta-analyses comparing the perioperative outcomes between LDLT and DDLT. Supplementary Table S8. Available results of the subgroup meta-analyses comparing the perioperative outcomes between LDLT and DDLT in recipient patients. Supplementary Table S9. Characteristics and quality assessment of the meta-analyses comparing the postoperative complications and retransplantation rate between LDLT and DDLT. Supplementary Table S10. Available results of the subgroup meta-analyses comparing the postoperative complications and retransplantation rate between LDLT and DDLT in recipient patients.

## Abbreviations

The following abbreviations are used in this manuscript:

LDLT	Living donor liver transplantation
DDLT	Deceased donor liver transplantation
LT	Liver transplantation
OPTN	Organ Procurement and Transplantation Network
SRTR	Scientific Registry of Transplant Recipients
ICU	Intensive Care Unit
PRISMA	Preferred Reporting Items for Systematic Reviews and Meta-Analyses
HCC	Hepatocellular carcinoma
DRO	Duration of the recipient operation
CIT	Cold ischemia time
RBC	Red blood cell
MELD	Model for end-stage liver disease
PELD	Pediatric End-Stage Liver Disease
HCV	Hepatitis C virus
