# Supplementary material for: Living Donor Liver Transplantation Versus Deceased Donor Liver Transplantation for Hepatocellular Carcinoma and HCV Patients: An Initial Umbrella Review
Source: J Clin Med. 2025 Apr 28;14(9):3047. doi: 10.3390/jcm14093047 (PMC12072381; doi:10.3390/jcm14093047)
Supplement: Supplementary file 1 [file jcm-14-03047-s001.zip › jcm-3509266-supplementary.pdf]

Supplementary Table S1. Characteristics and quality assessment of the meta-analyses comparing survival outcomes between LDLT and DDLT in recipient patients. Significant associations (P<0.05) are presented in bold. Associations reported in italic are those retained in the main analysis.

Outcomes	Author, year	Type of Subjects	No. of studies (T/R/C/P)	No. of LDLT/DDLT	MA metric	Effects model	Risk estimate(95%CI)	P-value	Favours	I^2; Q test P value	Egger test P value	AMSTAR 2 final rating
1-year OS	Zhu et al.[1],2019	Only HCC patients	14/0/14/0	862/1970	**RR**	**fixed**	**1.04[1.00 to 1.09]**	**0.03**	LDLT	36%;0.09	0.687	Critically low
1-year OS	Liang et al.[2],2012	Only HCC patients	5/0/5/0	233/559	OR	random	1.03[0.62 to 1.73]	0.9	Equivalent outcomes	0%;0.60	NPB	Critically low
3-year OS	Grant et al.[3],2013	Only HCC patients	12/0/12/0	637/1050	HR	fixed	0.97[0.73 to 1.27]	0.041	Equivalent outcomes	5.68%;NR	0.09	Critically low
3-year OS	Zhu et al.[1],2019	Only HCC patients	14/0/14/0	1057/2084	RR	random	1.03[0.96 to 1.11]	0.39	Equivalent outcomes	55%;0.007	0.687	Critically low
3-year OS	Liang et al.[2],2012	Only HCC patients	7/0/7/0	495/788	OR	random	1.07[0.77 to 1.48]	0.69	Equivalent outcomes	10%;0.35	NPB	Critically low
1-year OS	*Hu et al.[4], 2012*	*HCV-related diseases*	*9/0/9/0*	*231/931*	*OR*	*random*	*0.78[0.48 to 1.26]*	*0.31*	*Equivalent outcomes*	*0%;0.60*	*NPB*	*Moderate*
2-year OS	*Hu et al.[4], 2012*	*HCV-related diseases*	*6/0/6/0*	*127/509*	*OR*	*random*	*0.71[0.41 to 1.23]*	*0.23*	*Equivalent outcomes*	*0%;0.78*	*NPB*	*Moderate*

5-year OS	Zhu et al.[1],2019	Only HCC patients	17/0/17/0	957/3017	RR	random	1.04[0.95 to 1.13]	0.43	Equivalent outcomes	64%;0.0002	0.687	Critically low
5-year OS	Liang et al.[2],2012	Only HCC patients	4/0/4/0	162/578	OR	random	0.64[0.33 to 1.24]	0.18	Equivalent outcomes	47%;0.13	NPB	Critically low
3-year OS	*Hu et al.[4], 2012*	*HCV-related diseases*	*7/0/7/0*	*346/835*	*OR*	*random*	*0.79[0.55 to 1.12]*	*0.18*	*Equivalent outcomes*	*0%;0.70*	*NPB*	*Moderate*
5-year OS	Elkomos et al.[5],2023	Only HCC patients	21/0/21/0	2336/3744	RR	random	0.99[0.92 to 1.08]	0.89	Equivalent outcomes	75%;<0.00001	NPB	Critically low
4-year OS	*Hu et al.[4], 2012*	*HCV-related diseases*	*3/0/3/0*	*78/397*	*OR*	*random*	*0.92[0.43 to 1.95]*	*0.83*	*Equivalent outcomes*	*36%;0.21*	*NPB*	*Moderate*
5-year OS	*Hu et al.[4], 2012*	*HCV-related diseases*	*4/0/4/0*	*150/746*	*OR*	*random*	*1.06[0.53 to 2.14]*	*0.86*	*Equivalent outcomes*	*63%;0.04*	*NPB*	*Moderate*
1-year OS	*Barbetta et al.[6],2021*	*NR*	*19/0/19/0*	*4342/66205*	***HR***	***fixed***	***0.83[0.76 to 0.90]***	***<0.0001***	*LDLT*	*52%;0.007*	*NR*	*Critically low*
1-year ITT-OS	Zhu et al.[1],2019	Only HCC patients	2/0/2/0	142/554	RR	fixed	1.01[0.94 to 1.09]	0.81	Equivalent outcomes	0%;0.60	0.687	Critically low
3-year OS	*Barbetta et al.[6],2021*	*NR*	*17/0/17/0*	*4114/66157*	***HR***	***fixed***	***0.85[0.79 to 0.92]***	***<0.0001***	*LDLT*	*47%;0.02*	*NR*	*Critically low*
5-year OS	*Barbetta et al.[6],2021*	*NR*	*14/0/14/0*	*3892/65828*	***HR***	***fixed***	***0.87[0.81 to 0.93]***	***<0.0001***	*LDLT*	*52%;0.01*	*NR*	*Critically low*
3-year ITT-OS	Zhu et al.[1],2019	Only HCC patients	2/0/2/0	142/554	RR	fixed	1.08[0.97 to 1.21]	0.14	Equivalent outcomes	0%;0.71	0.687	Critically low

1-year OS	Elkomos et al.[5],2023	Only HCC patients	21/0/21/0	2380/3665	**RR**	**random**	**1.04[1.01 to 1.07]**	**0.01**	LDLT	46%;0.01	NPB	Critically low
2-year OS	Elkomos et al.[5],2023	Only HCC patients	14/0/14/0	1790/2320	RR	random	1.04[0.96 to 1.14]	0.33	Equivalent outcomes	78%; <0.00001	NPB	Critically low
5-year ITT-OS	Zhu et al.[1],2019	Only HCC patients	3/0/3/0	221/1174	**RR**	**fixed**	**1.11[1.01 to 1.22]**	**0.04**	LDLT	0%;0.69	0.687	Critically low
3-year OS	Elkomos et al.[5],2023	Only HCC patients	21/0/21/0	2336/3744	RR	random	0.99[0.92 to 1.08]	0.89	Equivalent outcomes	75%; <0.00001	NPB	Critically low
1-year DFS	Zhu et al.[1],2019	Only HCC patients	11/0/11/0	941/1235	RR	random	1.00[0.95 to 1.05]	0.99	Equivalent outcomes	59%;0.007	0.687	Critically low
4-year OS	Elkomos et al.[5],2023	Only HCC patients	12/0/12/0	1696/2121	**RR**	**random**	**1.09[1.02 to 1.17]**	**0.02**	LDLT	41%;0.07	NPB	Critically low
6-year OS	Elkomos et al.[5],2023	Only HCC patients	5/0/5/0	1327/675	RR	random	1.14[0.95 to 1.38]	0.16	Equivalent outcomes	83%;0.0001	NPB	Critically low
3-year DFS	Grant et al.[3],2013	Only HCC patients	12/0/12/0	633/1232	HR	random	1.59[1.02 to 2.49]	0.808	DDLT	50.07%;NR	0.23	Critically low
3-year DFS	Zhu et al.[1],2019	Only HCC patients	9/0/9/0	534/1124	RR	fixed	1.00[0.94 to 1.08]	0.89	Equivalent outcomes	35%;0.14	0.687	Critically low
10-year OS	Elkomos et al.[5],2023	Only HCC patients	2/0/2/0	962/429	RR	random	1.24[0.92 to 1.67]	0.16	Equivalent outcomes	90%;0.002	NPB	Critically low
1-year ITT-OS	Elkomos et al.[5],2023	Only HCC patients	5/0/5/0	641/2293	**RR**	**random**	**1.14[1.01 to 1.28]**	**0.03**	LDLT	88%; <0.00001	NPB	Critically low
2-year ITT-OS	Elkomos et al.[5],2023	Only HCC patients	3/0/3/0	303/1116	RR	random	1.23[1.00 to 1.50]	0.05	LDLT	85%;0.001	NPB	Critically low

3-year ITT-OS	*Elkomos et al.[5],2023*	*Only HCC patients*	*5/0/5/0*	*641/2293*	***RR***	***random***	***1.26[1.08 to 1.47]***	***0.004***	*LDLT*	*84%; <0.0001*	*NPB*	*Critically low*
4-year ITT-OS	*Elkomos et al.[5],2023*	*Only HCC patients*	*3/0/3/0*	*303/1116*	***RR***	***random***	***1.46[1.07 to 1.99]***	***0.02***	*LDLT*	*87%;0.0004*	*NPB*	*Critically low*
1-year OS	Cavalcante et al.[7],2022	NR	20/0/20/0	NR/NR	**HR**	**random**	**1.35[1.10 to 1.66]**	**0.005**	LDLT	73%;<0.00001	PB	Critically low
1-year OS	Tang et al.[8],2020	NR	18/0/18/0	2217/13193	**OR**	**random**	**1.32[1.01 to 1.72]**	**0.04**	LDLT	61%;0.0004	NPB	Critically low
3-year OS	Cavalcante et al.[7],2022	NR	21/0/21/0	NR/NR	**HR**	**random**	**1.26[1.09 to 1.46]**	**0.002**	LDLT	64%;<0.0001	PB	Critically low
3-year OS	Tang et al.[8],2020	NR	15/0/15/0	1851/9393	**OR**	**random**	**1.39[1.14 to 1.69]**	**0.001**	LDLT	41%;0.05	NPB	Critically low
5-year ITT-OS	*Elkomos et al.[5],2023*	*Only HCC patients*	*5/0/5/0*	*641/2293*	***RR***	***random***	***1.37[1.09 to 1.72]***	***0.006***	*LDLT*	*89%; <0.00001*	*NPB*	*Critically low*
1-year DFS	*Elkomos et al.[5],2023*	*Only HCC patients*	*14/0/14/0*	*2055/1923*	*RR*	*random*	*1.01[0.95 to 1.06]*	*0.79*	*Equivalent outcomes*	*66%;0.0002*	*NPB*	*Critically low*
2-year DFS	*Elkomos et al.[5],2023*	*Only HCC patients*	*6/0/6/0*	*555/727*	*RR*	*random*	*0.98[0.87 to 1.09]*	*0.69*	*Equivalent outcomes*	*60%;0.03*	*NPB*	*Critically low*
5-year OS	Cavalcante et al.[7],2022	NR	19/0/19/0	NR/NR	**HR**	**random**	**1.27[1.09 to 1.48]**	**0.002**	LDLT	72%;<0.00001	PB	Critically low
5-year OS	Tang et al.[8],2020	NR	16/0/16/0	1647/9759	**OR**	**random**	**1.33[1.04 to 1.70]**	**0.02**	LDLT	66%;<0.0001	NPB	Critically low
3-year DFS	*Elkomos et al.[5],2023*	*Only HCC patients*	*12/0/12/0*	*1831/1768*	*RR*	*random*	*1.00[0.92 to 1.09]*	*0.98*	*Equivalent outcomes*	*76%; <0.00001*	*NPB*	*Critically low*

5-year DFS	Zhu et al.[1],2019	*Only HCC patients*	*13/0/13/0*	*762/1625*	*RR*	*random*	*1.01[0.93 to 1.09]*	*0.85*	*Equivalent outcomes*	*58%;0.005*	*0.687*	*Critically low*
1-year RFS	*Liang et al.[2],2012*	*Only HCC patients*	*3/0/3/0*	*333/463*	*OR*	*random*	*0.86[0.54 to 1.38]*	*0.54*	*Equivalent outcomes*	*0%;0.58*	*NPB*	*Critically low*
3-year RFS	*Liang et al.[2],2012*	*Only HCC patients*	*4/0/4/0*	*179/576*	*OR*	*random*	*1.04[0.69 to 1.58]*	*0.84*	*Equivalent outcomes*	*0%;0.82*	*NPB*	*Critically low*
5-year RFS	*Liang et al.[2],2012*	*Only HCC patients*	*3/0/3/0*	*121/542*	*OR*	*random*	*1.11[0.70 to 1.77]*	*0.65*	*Equivalent outcomes*	*0%;0.65*	*NPB*	*Critically low*

MA, meta-analysis; CI, confidence interval; T, total No. of studies; R, randomized controlled trial; C, cohort studies; P, population-based case-control and/or cross-sectional studies; RR, relative risk; OR, odds ratio; HR, hazard ratio; NR, not reported; OS, overall survival; ITT-OS, intention-to-treat overall survival; DFS, disease-free survival; RFS, recurrence-free survival; LDLT, living donor liver transplantation; DDLT, deceased donor liver transplantation; NPB, no evidence of publication bias; PB, publication bias.

Supplementary Table S2. Available results of the subgroup meta-analyses comparing survival outcomes between LDLT and DDLT in recipient patients. Significant associations (P<0.05) are presented in bold.

Subgroup analysis	Classification	Author, year	Diagnosis of the patients	No. of studies	MA metric	Risk estimate[95%CI]	P-value	Favours	I^2; Q test P value	AMSTAR final rating
**1-year OS**										

**Milan criteria**	Within Milan criteria	Zhu et al.[1],2019	Only HCC patients	3	RR	1.08[0.94 to 1.24]	0.29	Equivalent outcomes	77%; 0.01	Critically low
	Within Milan criteria	Liang et al.[2],2012	Only HCC patients	3	OR	1.02[0.45 to 2.35]	0.96	Equivalent outcomes	0%; 0.76	Critically low
	Within Milan criteria	Elkomos et al.[5],2023	Only HCC patients	5	RR	1.04[0.96 to 1.12]	0.31	Equivalent outcomes	72%; 0.006	Critically low
	Beyond Milan criteria	Liang et al.[2],2012	Only HCC patients	3	OR	0.74[0.35 to 1.61]	0.45	Equivalent outcomes	0%; 0.98	Critically low
	Beyond Milan criteria	Elkomos et al.[5],2023	Only HCC patients	4	RR	1.02[0.94 to 1.10]	0.62	Equivalent outcomes	0%; 0.73	Critically low
**Sample size**	Sample size ≥300	Zhu et al.[1],2019	Only HCC patients	4	**RR**	**1.08[1.01 to 1.15]**	**0.03**	LDLT	64%; 0.04	Critically low
	Sample size < 300	Zhu et al.[1],2019	Only HCC patients	10	RR	1.02[0.97 to 1.07]	0.53	Equivalent outcomes	0%; 0.45	Critically low
	Sample size ＜100	Hu et al.[4], 2012	HCV-related diseases	5	OR	1.06[0.32 to 3.57]	0.92	Equivalent outcomes	0%; 0.67	Moderate
	Sample size ≥ 100	Hu et al.[4], 2012	HCV-related diseases	4	OR	0.75[0.41 to 1.36]	0.34	Equivalent outcomes	19%; 0.29	Moderate
	LDLT < 100	Tang et al.[8],2020	NR	9	OR	1.27[0.90 to 1.77]	0.17	Equivalent outcomes	33%; 0.15	Critically low
	LDLT ≥ 100	Tang et al.[8],2020	NR	9	**OR**	**1.43[1.02 to 2.01]**	**0.04**	LDLT	74%; 0.0002	Critically low
**Diagnosis of the patients**	HCC related	Tang et al.[8],2020	Only HCC patients	9	**OR**	**1.68[1.19 to 2.37]**	**0.003**	LDLT	49%; 0.05	Critically low
	Not HCC related	Tang et al.[8],2020	Not HCC related	9	OR	1.07[0.87 to 1.32]	0.5	Equivalent outcomes	35%; 0.14	Critically low

**Region**	Asia	Zhu et al.[1],2019	Only HCC patients	6	RR	1.08[1.00 to 1.17]	0.05	Equivalent outcomes	51%; 0.07	Critically low
	Asia	Elkomos et al.[5],2023	Only HCC patients	11	RR	1.03[0.98 to 1.07]	0.25	Equivalent outcomes	44%; 0.06	Critically low
	America	Zhu et al.[1],2019	Only HCC patients	6	RR	1.04[0.99 to 1.09]	0.1	Equivalent outcomes	39%; 0.14	Critically low
	America	Elkomos et al.[5],2023	Only HCC patients	8	**RR**	**1.06[1.02 to 1.10]**	**0.002**	LDLT	31%; 0.18	Critically low
	Europe	Elkomos et al.[5],2023	Only HCC patients	5	RR	0.99[0.91 to 1.09]	0.9	Equivalent outcomes	44%; 0.13	Critically low
**Perdominant sample proportion**	LDLT	Liang et al.[2],2012	Only HCC patients	2	OR	2.00[0.72 to 5.53]	0.18	Equivalent outcomes	0%; 0.84	Critically low
	DDLT	Liang et al.[2],2012	Only HCC patients	3	OR	0.82[0.45 to 1.50]	0.52	Equivalent outcomes	0%; 0,78	Critically low
	Ratio of LDLT/DDLT＞0.5	Hu et al.[4], 2012	HCV-related diseases	3	OR	0.94[0.31 to 2.84]	0.91	Equivalent outcomes	0%; 0.63	Moderate
	Ratio of LDLT/DDLT＜0.5	Hu et al.[4], 2012	HCV-related diseases	6	OR	0.76[0.42 to 1.36]	0.35	Equivalent outcomes	8%; 0.37	Moderate
**Study design**	Prospective studies	Hu et al.[4], 2012	HCV-related diseases	3	OR	0.70[0.33 to 1.46]	0.34	Equivalent outcomes	0%; 0.76	Moderate
	Retrospective studies	Hu et al.[4], 2012	HCV-related diseases	6	OR	0.91[0.44 to 1.87]	0.8	Equivalent outcomes	13%; 0.33	Moderate
**Presence of HCC**	More in LDLT group	Hu et al.[4], 2012	HCV-related diseases	4	OR	0.48[0.21 to 1.13]	0.09	Equivalent outcomes	0%; 0.44	Moderate

	More in DDLT group	Hu et al.[4], 2012	HCV-related diseases	4	OR	1.02[0.56 to 1.87]	0.94	Equivalent outcomes	0%; 0.67	Moderate
**2-year OS**										
**Milan criteria**	Within Milan criteria	Elkomos et al.[5],2023	Only HCC patients	4	RR	1.06[0.97 to 1.16]	0.22	Equivalent outcomes	70%; 0.02	Critically low
	Beyond Milan criteria	Elkomos et al.[5],2023	Only HCC patients	4	RR	1.06[0.95 to 1.18]	0.33	Equivalent outcomes	0%; 0.83	Critically low
**Region**	Asia	Elkomos et al.[5],2023	Only HCC patients	5	RR	1.07[1.00 to 1.14]	0.07	Equivalent outcomes	0%; 0.56	Critically low
	America	Elkomos et al.[5],2023	Only HCC patients	5	RR	1.01[0.89 to 1.15]	0.83	Equivalent outcomes	67%; 0.02	Critically low
	Europe	Elkomos et al.[5],2023	Only HCC patients	3	RR	0.88[0.64 to 1.22]	0.44	Equivalent outcomes	85%; 0.001	Critically low
**Study design**	Prospective studies	Hu et al.[4], 2012	HCV-related diseases	2	OR	0.65[0.18 to 2.39]	0.52	Equivalent outcomes	0%; 0.97	Moderate
	Retrospective studies	Hu et al.[4], 2012	HCV-related diseases	4	OR	0.73[0.40 to 1.33]	0.3	Equivalent outcomes	0%; 0.48	Moderate
**Sample size**	Sample size ＜100	Hu et al.[4], 2012	HCV-related diseases	4	OR	0.79[0.33 to 1.85]	0.58	Equivalent outcomes	0%; 0.98	Moderate
	Sample size ≥ 100	Hu et al.[4], 2012	HCV-related diseases	2	OR	0.68[0.23 to 2.00]	0.49	Equivalent outcomes	55%; 0.14	Moderate
**Median year of follow-up period**	2001 or earlier	Hu et al.[4], 2012	HCV-related diseases	3	OR	0.70[0.35 to 1.41]	0.32	Equivalent outcomes	13%; 0.32	Moderate
	After 2001	Hu et al.[4], 2012	HCV-related diseases	3	OR	0.77[0.27 to 2.15]	0.61	Equivalent outcomes	0%; 0.92	Moderate

**Presence of HCC**	More in LDLT group	Hu et al.[4], 2012	HCV-related diseases	3	OR	0.52[0.25 to 1.10]	0.09	Equivalent outcomes	0%; 0.71	Moderate
	More in DDLT group	Hu et al.[4], 2012	HCV-related diseases	2	OR	1.15[0.47 to 2.83]	0.76	Equivalent outcomes	0%; 0.86	Moderate
**Perdominant sample proportion**	Ratio of LDLT/DDLT＞0.5	Hu et al.[4], 2012	HCV-related diseases	3	OR	0.98[0.44 to 2.16]	0.96	Equivalent outcomes	0%; 0.84	Moderate
	Ratio of LDLT/DDLT＜0.5	Hu et al.[4], 2012	HCV-related diseases	3	OR	0.53[0.25 to 1.13]	0.1	Equivalent outcomes	0%; 0.64	Moderate
**3-year OS**										
**Milan criteria**	Within Milan criteria	Zhu et al.[1],2019	Only HCC patients	4	RR	1.02[0.94 to 1.10]	0.63	Equivalent outcomes	0%; 0.97	Critically low
	Within Milan criteria	Liang et al.[2],2012	Only HCC patients	5	OR	0.95[0.57 to 1.58]	0.83	Equivalent outcomes	0%; 0.53	Critically low
	Within Milan criteria	Elkomos et al.[5],2023	Only HCC patients	5	RR	1.01[0.88 to 1.16]	0.88	Equivalent outcomes	81%; 0.0002	Critically low
	Beyond Milan criteria Milan criteria	Zhu et al.[1],2019	Only HCC patients	2	RR	1.09[0.80 to 1.47]	0.59	Equivalent outcomes	56%; 0.13	Critically low
	Beyond Milan criteria Milan criteria	Liang et al.[2],2012	Only HCC patients	4	OR	1.07[0.62 to 1.83]	0.82	Equivalent outcomes	0%; 0.49	Critically low
	Beyond Milan criteria Milan criteria	Elkomos et al.[5],2023	Only HCC patients	4	**RR**	**1.16[1.01 to 1.32]**	**0.03**	LDLT	0%; 0.6	Critically low

**UCSF criteria**	Within UCSF criteria	Zhu et al.[1],2019	Only HCC patients	3	RR	1.08[0.98 to 1.20]	0.13	Equivalent outcomes	0%; 0.38	Critically low
	Beyond Milan criteria UCSF criteria	Zhu et al.[1],2019	Only HCC patients	2	RR	0.75[0.27 to 2.04]	0.57	Equivalent outcomes	0%;0.88	Critically low
**Sample size**	Sample size ≥300	Zhu et al.[1],2019	Only HCC patients	5	RR	1.10[0.99 to 1.21]	0.07	Equivalent outcomes	59%; 0.04	Critically low
	Sample size < 300	Zhu et al.[1],2019	Only HCC patients	9	RR	0.97[0.90 to 1.05]	0.45	Equivalent outcomes	15%; 0.31	Critically low
	LDLT < 100	Tang et al.[8],2020	NR	8	OR	1.17[0.87 to 1.58]	0.29	Equivalent outcomes	0%; 0.70	Critically low
	LDLT ≥ 100	Tang et al.[8],2020	NR	7	**OR**	**1.52[1.18 to 1.95]**	**0.001**	LDLT	60%; 0.02	Critically low
**Diagnosis of the patients**	HCC related	Tang et al.[8],2020	Only HCC patients	9	**OR**	**1.55[1.17 to 2.04]**	**0.002**	LDLT	43%; 0.08	Critically low
	Not HCC related	Tang et al.[8],2020	Not HCC related	6	**OR**	**1.21[0.98 to 1.50]**	**0.07**	LDLT	0%; 0.75	Critically low
**Region**	Asia	Zhu et al.[1],2019	Only HCC patients	6	RR	1.11[0.95 to 1.30]	0.19	Equivalent outcomes	82%; 0.0001	Critically low
	Asia	Elkomos et al.[5],2023	Only HCC patients	8	RR	1.09[0.98 to 1.21]	0.1	Equivalent outcomes	68%; 0.003	Critically low
	America	Zhu et al.[1],2019	Only HCC patients	7	RR	1.04[0.94 to 1.15]	0.48	Equivalent outcomes	69%; 0.003	Critically low
	America	Elkomos et al.[5],2023	Only HCC patients	6	RR	1.05[0.93 to 1.18]	0.45	Equivalent outcomes	77%; 0.0005	Critically low
	Europe	Zhu et al.[1],2019	Only HCC patients	2	RR	0.92[0.77 to 1.11]	0.4	Equivalent outcomes	0%; 0.98	Critically low

	Europe	Elkomos et al.[5],2023	Only HCC patients	4	RR	1.03[0.95 to 1.13]	0.46	Equivalent outcomes	0%; 0.72	Critically low
**Perdominant sample proportion**	LDLT	Liang et al.[2],2012	Only HCC patients	3	OR	1.28[0.64 to 2.55]	0.48	Equivalent outcomes	38%; 0.20	Critically low
	DDLT	Liang et al.[2],2012	Only HCC patients	4	OR	0.87[0.58 to 1.30]	0.49	Equivalent outcomes	0%; 0.85	Critically low
	Ratio of LDLT/DDLT＞0.5	Hu et al.[4], 2012	HCV-related diseases	3	OR	0.79[0.48 to 1.30]	0.36	Equivalent outcomes	0%; 0.40	Moderate
	Ratio of LDLT/DDLT＜0.5	Hu et al.[4], 2012	HCV-related diseases	4	OR	0.78[0.47 to 1.29]	0.33	Equivalent outcomes	0%; 0.58	Moderate
**Study design**	Prospective studies	Hu et al.[4], 2012	HCV-related diseases	3	OR	0.65[0.18 to 2.39]	0.22	Equivalent outcomes	0%; 0.71	Moderate
	Retrospective studies	Hu et al.[4], 2012	HCV-related diseases	4	OR	0.85[0.47 to 1.53]	0.58	Equivalent outcomes	1%; 0.39	Moderate
**Sample size**	Sample size ＜100	Hu et al.[4], 2012	HCV-related diseases	3	OR	0.98[0.42 to 2.29]	0.95	Equivalent outcomes	0%; 0.90	Moderate
	Sample size ≥ 100	Hu et al.[4], 2012	HCV-related diseases	4	OR	0.76[0.50 to 1.14]	0.18	Equivalent outcomes	9%; 0.35	Moderate
**Median year of follow-up period**	2001 or earlier	Hu et al.[4], 2012	HCV-related diseases	3	OR	0.80[0.38 to 1.67]	0.55	Equivalent outcomes	25%; 0.26	Moderate
	After 2001	Hu et al.[4], 2012	HCV-related diseases	4	OR	0.79[0.51 to 1.21]	0.27	Equivalent outcomes	0%; 0.77	Moderate
**Presence of HCC**	More in LDLT group	Hu et al.[4], 2012	HCV-related diseases	2	OR	0.56[0.26 to 1.23]	0.15	Equivalent outcomes	0%; 0.42	Moderate

	More in DDLT group	Hu et al.[4], 2012	HCV-related diseases	3	OR	1.09[0.63 to 1.89]	0.75	Equivalent outcomes	0%; 0.76	Moderate
**4-year OS**										
**Milan criteria**	Within Milan criteria	Elkomos et al.[5],2023	Only HCC patients	4	RR	1.07[0.92 to 1.25]	0.39	Equivalent outcomes	83%; 0.0005	Critically low
	Beyond Milan criteria Milan criteria	Elkomos et al.[5],2023	Only HCC patients	4	**RR**	**1.20[1.04 to 1.38]**	**0.01**	LDLT	32%; 0.22	Critically low
**Region**	Asia	Elkomos et al.[5],2023	Only HCC patients	5	RR	1.08[1.00 to 1.18]	0.06	Equivalent outcomes	0%; 0.81	Critically low
	America	Elkomos et al.[5],2023	Only HCC patients	3	RR	1.11[0.93 to 1.34]	0.26	Equivalent outcomes	54%; 0.11	Critically low
	Europe	Elkomos et al.[5],2023	Only HCC patients	3	RR	0.99[0.90 to 1.10]	0.86	Equivalent outcomes	0%; 0.50	Critically low
**Study design**	Retrospective studies	Hu et al.[4], 2012	HCV-related diseases	2	OR	0.96[0.29 to 3.21]	0.94	Equivalent outcomes	68%; 0.08	Moderate
**Sample size**	Sample size ≥ 100	Hu et al.[4], 2012	HCV-related diseases	2	OR	0.96[0.29 to 3.21]	0.94	Equivalent outcomes	68%; 0.08	Moderate
**Median year of follow-up period**	2001 or earlier	Hu et al.[4], 2012	HCV-related diseases	2	OR	0.96[0.29 to 3.21]	0.89	Equivalent outcomes	86%; 0.008	Moderate
**Perdominant sample proportion**	Ratio of LDLT/DDLT＜0.5	Hu et al.[4], 2012	HCV-related diseases	2	OR	0.63[0.29 to 1.33]	0.22	Equivalent outcomes	0%; 0.54	Moderate
**5-year OS**										
**Milan criteria**	Within Milan criteria	Zhu et al.[1],2019	Only HCC patients	2	RR	1.03[0.87 to 1.21]	0.77	Equivalent outcomes	0%; 0.42	Critically low

	Within Milan criteria	Liang et al.[2],2012	Only HCC patients	4	OR	0.74[0.30 to 1.82]	0.51	Equivalent outcomes	62%; 0.05	Critically low
	Within Milan criteria	Elkomos et al.[5],2023	Only HCC patients	5	RR	1.10[0.93 to 1.29]	0.27	Equivalent outcomes	83%; 0.0001	Critically low
	Beyond Milan criteria Milan criteria	Liang et al.[2],2012	Only HCC patients	2	OR	0.92[0.07 to 12.34]	0.95	Equivalent outcomes	83%; 0.02	Critically low
	Beyond Milan criteria Milan criteria	Elkomos et al.[5],2023	Only HCC patients	3	**RR**	**1.32[1.13 to 1.54]**	**0.0006**	LDLT	0%; 0.79	Critically low
**Sample size**	Sample size ≥300	Zhu et al.[1],2019	Only HCC patients	5	RR	1.07[0.92 to 1.25]	0.38	Equivalent outcomes	80%; 0.005	Critically low
	Sample size < 300	Zhu et al.[1],2019	Only HCC patients	12	RR	1.01[0.91 to 1.12]	0.83	Equivalent outcomes	44%; 0.05	Critically low
	LDLT < 100	Tang et al.[8],2020	NR	10	OR	1.20[0.95 to 1.52]	0.13	Equivalent outcomes	24%; 0.22	Critically low
	LDLT ≥ 100	Tang et al.[8],2020	NR	6	**OR**	**1.49[1.02 to 2.16]**	**0.04**	LDLT	81%; < 0.0001	Critically low
**Diagnosis of the patients**	HCC related	Tang et al.[8],2020	Only HCC patients	10	OR	1.43[1.01 to 2.03]	0.05	Equivalent outcomes	0.0003, I 2 = 71%	Critically low
	Not HCC related	Tang et al.[8],2020	Not HCC related	6	OR	1.15[0.92 to 1.42]	0.21	Equivalent outcomes	0%; 0.64	Critically low
**Region**	Asia	Zhu et al.[1],2019	Only HCC patients	6	RR	1.11[0.99 to 1.25]	0.09	Equivalent outcomes	25%; 0.25	Critically low

	Asia	Elkomos et al.[5],2023	Only HCC patients	9	RR	1.03[0.95 to 1.12]	0.41	Equivalent outcomes	22%; 0.25	Critically low
	America	Zhu et al.[1],2019	Only HCC patients	7	RR	1.04[0.98 to 1.11]	0.22	Equivalent outcomes	83%; < 0.0001	Critically low
	America	Elkomos et al.[5],2023	Only HCC patients	5	RR	1.02[0.90 to 1.16]	0.76	Equivalent outcomes	68%; 0.01	Critically low
	Europe	Zhu et al.[1],2019	Only HCC patients	3	RR	0.97[0.86 to 1.09]	0.6	Equivalent outcomes	0%; 0.71	Critically low
	Europe	Elkomos et al.[5],2023	Only HCC patients	4	RR	0.87[0.71 to 1.06]	0.18	Equivalent outcomes	58%; 0.07	Critically low
**Perdominant sample proportion**	DDLT	Liang et al.[2],2012	Only HCC patients	3	OR	0.80[0.51 to 1.26]	0.34	Equivalent outcomes	0%;0.48	Critically low
	Ratio of LDLT/DDLT＜0.5	Hu et al.[4], 2012	HCV-related diseases	3	OR	0.86[0.40 to 1.88]	0.71	Equivalent outcomes	63%; 0.07	Moderate
**Study design**	Retrospective studies	Hu et al.[4], 2012	HCV-related diseases	3	OR	1.07[0.37 to 3.15]	0.9	Equivalent outcomes	75%; 0.02	Moderate
**Sample size**	Sample size ≥ 100	Hu et al.[4], 2012	HCV-related diseases	4	OR	1.06[0.53 to 2.14]	0.86	Equivalent outcomes	63%; 0.04	Moderate
**Median year of follow-up period**	2001 or earlier	Hu et al.[4], 2012	HCV-related diseases	2	OR	0.89[0.15 to 5.20]	0.13	Equivalent outcomes	26%; 0.16	Moderate
	After 2001	Hu et al.[4], 2012	HCV-related diseases	2	OR	1.21[0.71 to 2.06]	0.49	Equivalent outcomes	0%; 0.48	Moderate
**Presence of HCC**	More in DDLT group	Hu et al.[4], 2012	HCV-related diseases	3	OR	1.39[0.87 to 2.21]	0.17	Equivalent outcomes	0%; 0.45	Moderate
**6-year OS**										

**Milan criteria**	Within Milan criteria	Elkomos et al.[5],2023	Only HCC patients	3	RR	1.22[0.97 to 1.52]	0.09	Equivalent outcomes	88%; 0.0002	Critically low
	Beyond Milan criteria Milan criteria	Elkomos et al.[5],2023	Only HCC patients	2	RR	1.30[1.03 to 1.64]	0.02	Equivalent outcomes	0%; 0.75	Critically low
**Region**	Asia	Elkomos et al.[5],2023	Only HCC patients	2	RR	1.06[0.95 to 1.18]	0.33	Equivalent outcomes	0%; 0.83	Critically low
	America	Elkomos et al.[5],2023	Only HCC patients	2	RR	1.17[0.61 to 2.23]	0.63	Equivalent outcomes	93%; 0.0002	Critically low
**10-year OS**										
**Milan criteria**	Within Milan criteria	Elkomos et al.[5],2023	Only HCC patients	2	RR	1.23[0.83 to 1.84]	0.3	Equivalent outcomes	96%; < 0.00001	Critically low
	Beyond Milan criteria Milan criteria	Elkomos et al.[5],2023	Only HCC patients	2	**RR**	**1.42[1.07 to 1.87]**	**0.01**	LDLT	34%; 0.22	Critically low
**1-year DFS**										
**Sample size**	Sample size ≥300	Zhu et al.[1],2019	Only HCC patients	3	RR	1.04[0.93 to 1.18]	0.47	Equivalent outcomes	74%; 0.02	Critically low
	Sample size < 300	Zhu et al.[1],2019	Only HCC patients	8	RR	0.98[0.93 to 1.04]	0.5	Equivalent outcomes	51%; 0.05	Critically low
**Region**	Asia	Zhu et al.[1],2019	Only HCC patients	7	RR	1.01[0.93 to 1.10]	0.75	Equivalent outcomes	66%; 0.007	Critically low
	Asia	Elkomos et al.[5],2023	Only HCC patients	9	RR	1.02[0.95 to 1.09]	0.6	Equivalent outcomes	69%; 0.001	Critically low

	America	Zhu et al.[1],2019	Only HCC patients	3	RR	1.00[0.89 to 1.12]	0.97	Equivalent outcomes	78%; 0.01	Critically low
	America	Elkomos et al.[5],2023	Only HCC patients	5	RR	0.99[0.89 to 1.09]	0.78	Equivalent outcomes	68%; 0.01	Critically low
**Milan criteria**	Within Milan criteria	Elkomos et al.[5],2023	Only HCC patients	2	RR	0.99[0.89 to 1.10]	0.86	Equivalent outcomes	64%; 0.10	Critically low
**2-year DFS**										
**Region**	Asia	Elkomos et al.[5],2023	Only HCC patients	3	RR	1.07[0.87 to 1.31]	0.54	Equivalent outcomes	78%; 0.01	Critically low
	America	Elkomos et al.[5],2023	Only HCC patients	3	**RR**	**0.89[0.80 to 0.99]**	**0.03**	DDLT	0%; 0.95	Critically low
**3-year DFS**										
**Milan criteria**	Within Milan criteria	Zhu et al.[1],2019	Only HCC patients	2	RR	0.95[0.76 to 1.19]	0.66	Equivalent outcomes	47%; 0.17	Critically low
	Within Milan criteria	Elkomos et al.[5],2023	Only HCC patients	2	**RR**	**0.93[0.89 to 0.97]**	**0.0006**	DDLT	6%; 0.30	Critically low
**UCSF criteria**	Within UCSF criteria	Zhu et al.[1],2019	Only HCC patients	2	RR	0.85[0.44 to 1.64]	0.62	Equivalent outcomes	86%; 0.008	Critically low
**Sample size**	Sample size ≥300	Zhu et al.[1],2019	Only HCC patients	2	RR	1.07[0.93 to 1.22]	0.35	Equivalent outcomes	0%; 0.37	Critically low
	Sample size < 300	Zhu et al.[1],2019	Only HCC patients	7	RR	0.99[0.91 to 1.08]	0.85	Equivalent outcomes	52%; 0.05	Critically low
**Region**	Asia	Zhu et al.[1],2019	Only HCC patients	3	RR	1.01[0.85 to 1.20]	0.89	Equivalent outcomes	52%; 0.13	Critically low

	Asia	Elkomos et al.[5],2023	Only HCC patients	7	RR	1.00[0.88 to 1.14]	0.98	Equivalent outcomes	79%; < 0.0001	Critically low
	America	Zhu et al.[1],2019	Only HCC patients	4	RR	0.95[0.86 to 1.05]	0.14	Equivalent outcomes	25%; 0.26	Critically low
	America	Elkomos et al.[5],2023	Only HCC patients	4	RR	0.99[0.84 to 1.16]	0.85	Equivalent outcomes	74%; 0.008	Critically low
	Europe	Zhu et al.[1],2019	Only HCC patients	2	RR	1.08[1.00 to 1.18]	0.63	Equivalent outcomes	0%; 0.41	Critically low
**4-year DFS**										
**Region**	Asia	Elkomos et al.[5],2023	Only HCC patients	3	RR	1.04[0.95 to 1.15]	0.39	Equivalent outcomes	0%; 0.53	Critically low
	America	Elkomos et al.[5],2023	Only HCC patients	2	**RR**	**0.77[0.64 to 0.91]**	**0.003**	DDLT	0%; 0.86	Critically low
**5-year DFS**										
**Sample size**	Sample size ≥300	Zhu et al.[1],2019	Only HCC patients	2	RR	1.08[0.93 to 1.26]	0.31	Equivalent outcomes	0%; 0.38	Critically low
	Sample size < 300	Zhu et al.[1],2019	Only HCC patients	11	RR	0.98[0.93 to 1.04]	0.59	Equivalent outcomes	62%; 0.003	Critically low
**Region**	Asia	Zhu et al.[1],2019	Only HCC patients	6	RR	0.98[0.85 to 1.14]	0.84	Equivalent outcomes	69%; 0.006	Critically low
	Asia	Elkomos et al.[5],2023	Only HCC patients	10	RR	1.00[0.93 to 1.08]	1	Equivalent outcomes	38%; 0.11	Critically low
	America	Zhu et al.[1],2019	Only HCC patients	5	RR	0.99[0.89 to 1.10]	0.87	Equivalent outcomes	37%; 0.18	Critically low

	America	Elkomos et al.[5],2023	Only HCC patients	3	RR	0.97[0.79 to 1.19]	0.78	Equivalent outcomes	78%; 0.01	Critically low
	Europe	Zhu et al.[1],2019	Only HCC patients	2	RR	1.13[0.97 to 1.31]	0.12	Equivalent outcomes	57%; 0.13	Critically low
	Europe	Elkomos et al.[5],2023	Only HCC patients	3	RR	1.06[0.98 to 1.14]	0.18	Equivalent outcomes	0%; 0.55	Critically low
**Milan criteria**	Within Milan criteria	Elkomos et al.[5],2023	Only HCC patients	2	RR	0.96[0.83 to 1.11]	0.54	Equivalent outcomes	47%; 0.17	Critically low
**1-year RFS**										
**Milan criteria**	Beyond Milan criteria Milan criteria	Liang et al.[2],2012	Only HCC patients	2	OR	1.02[0.42 to 2.49]	0.97	Equivalent outcomes	21%; 0.26	Critically low
**Perdominant sample proportion**	DDLT	Liang et al.[2],2012	Only HCC patients	2	OR	0.98[0.54 to 1.77]	0.95	Equivalent outcomes	0%; 0.42	Critically low
**3-year RFS**										
**Milan criteria**	Within Milan criteria	Liang et al.[2],2012	Only HCC patients	2	OR	0.91[0.26 to 3.23]	0.89	Equivalent outcomes	40%; 0.20	Critically low
	Beyond Milan criteria Milan criteria	Liang et al.[2],2012	Only HCC patients	2	OR	1.30[0.66 to 2.57]	0.45	Equivalent outcomes	4%; 0.31	Critically low
**Perdominant sample proportion**	DDLT	Liang et al.[2],2012	Only HCC patients	3	OR	1.10[0.68 to 1.78]	0.7	Equivalent outcomes	0%; 0.69	Critically low
**5-year RFS**										
**Milan criteria**	Within Milan criteria	Liang et al.[2],2012	Only HCC patients	2	OR	1.10[0.55 to 2.20]	0.79	Equivalent outcomes	0%; 0.34	Critically low

	Beyond Milan criteria Milan criteria	Liang et al.[2],2012	Only HCC patients	2	OR	1.03[0.4 to 1.96]	0.92	Equivalent outcomes	0%; 0.92	Critically low
**Perdominant sample proportion**	DDLT	Liang et al.[2],2012	Only HCC patients	3	OR	1.11[0.70 to 1.77]	0.65	Equivalent outcomes	0%;0.65	Critically low

MA, meta-analysis; CI, confidence interval; RR, relative risk; OR, odds ratio; HR, hazard ratio; NR, not reported; OS, overall survival; ITT-OS, intention-to-treat overall survival; DFS, disease-free survival; RFS, recurrence-free survival; LDLT, living donor liver transplantation; DDLT, deceased donor liver transplantation.

Supplementary Table S3. Characteristics and quality assessment of the meta-analyses comparing the disease relapse between LDLT and DDLT in recipient patients. Significant associations (P<0.05) are presented in bold. Associations reported in italic are those retained in the main analysis.

Outcomes	Author, year	Type of Subjects	No. of studies (T/R/C/P)	No. of LDLT/DDLT	MA metric	Effects model	Risk estimate(95%CI)	P-value	Favours	I^2; Q test P value	Egger test P value	AMSTAR 2 final rating
1-year HCC recurrence rates	Zhu et al.[1],2019	Only HCC patients	2/0/2/0	178/417	RR	fixed	1.41[0.72 to 2.77]	0.32	Equivalent outcomes	0%;0.36	0.687	Critically low
1-year HCC recurrence rates	*Tang et al.[8],2020*	*Only HCC patients*	*8/0/8/0*	*1201/7480*	*OR*	*random*	*1.00[0.61 to 1.66]*	*0.99*	*Equivalent outcomes*	*72%;0.0007*	*PB*	*Critically low*
1-year HCC recurrence rates	Liang et al.[2],2012	Only HCC patients	4/0/4/0	190/448	OR	random	1.55[0.36 to 6.58]	0.55	Equivalent outcomes	40%;0.17	NPB	Critically low

3-year HCC recurrence rates	Zhu et al.[1],2019	Only HCC patients	2/0/2/0	178/417	RR	fixed	0.89[0.57 to 1.39]	0.6	Equivalent outcomes	0%;0.41	0.687	Critically low
3-year HCC recurrence rates	Tang et al.[8],2020	Only HCC patients	5/0/5/0	751/7261	OR	random	0.86[0.52 to 1.41]	0.54	Equivalent outcomes	78%;0.001	PB	Critically low
3-year HCC recurrence rates	Liang et al.[2],2012	Only HCC patients	4/0/4/0	190/448	OR	random	2.57[0.53 to 12.41]	0.24	Equivalent outcomes	72%;0.01	NPB	Critically low
5-year HCC recurrence rates	Zhu et al.[1],2019	Only HCC patients	4/0/4/0	291/1183	RR	random	0.85[0.56 to 1.31]	0.47	Equivalent outcomes	40%;0.17	0.687	Critically low
5-year HCC recurrence rates	Tang et al.[8],2020	Only HCC patients	8/0/8/0	1036/7981	OR	random	0.87[0.54 to 1.38]	0.55	Equivalent outcomes	81%; <0.00001	NPB	Critically low
5-year HCC recurrence rates	Liang et al.[2],2012	Only HCC patients	3/0/3/0	132/414	OR	random	1.21[0.44 to 3.32]	0.71	Equivalent outcomes	44%;0.17	NPB	Critically low
HCC recurrence rates	Elkomos et al.[5],2023	Only HCC patients	16/0/16/0	1214/2403	RR	random	1.07[0.77 to 1.48]	0.7	Equivalent outcomes	62%;0.0005	NPB	Critically low
Accumulated HCC recurrence rates	Zhang et al.[9],2019	Only HCC patients	7/0/7/0	527/781	**HR**	**NR**	**1.51[1.09 to 2.11]**	**0.01**	DDLT	48%;0.07	NR	Critically low
HCV Recurrence rates	Tang et al.[8],2020	HCV-related diseases	4/0/4/0	142/664	OR	random	1.10[0.39 to 3.10]	0.86	Equivalent outcomes	81%;0.001	NPB	Critically low
HCV Recurrence rates	Hu et al.[4], 2012	HCV-related diseases	7/0/7/0	175/575	OR	random	0.78[0.35 to 1.72]	0.53	Equivalent outcomes	71%;0.002	NPB	Moderate

MA, meta-analysis; CI, confidence interval; T, total No. of studies; R, randomized controlled trial; C, cohort studies; P, population-based case-control and/or cross-sectional studies; RR, relative risk; OR, odds ratio; HR, hazard ratio; NR, not reported; LDLT, living donor liver transplantation; DDLT, deceased donor liver transplantation; NPB, no evidence of publication bias; PB, publication bias.

Supplementary Table S4. Available results of the subgroup meta-analyses comparing the disease relapse between LDLT and DDLT in recipient patients. Significant associations (P<0.05) are presented in bold.

Subgroup analysis	Classification	Author, year	Diagnosis of the patients	No. of studies	MA metric	Risk estimate[95%CI]	P-value	Favours	I^2; Q test P value	AMSTAR final rating
**HCV recurrence**										
**Sample size**	DDLT < 100	Tang et al.[8],2020	HCV-related diseases	2	OR	1.92[0.95 to 3.87]	0.07	Equivalent outcomes	39%; 0.20	Critically low
	DDLT ≥ 100	Tang et al.[8],2020	HCV-related diseases	2	OR	0.62[0.12 to 3.15]	0.56	Equivalent outcomes	87%; 0.006	Critically low
	Sample size ＜100	Hu et al.[4], 2012	HCV-related diseases	4	OR	0.63[0.25 to 1.57]	0.32	Equivalent outcomes	47%; 0.13	Moderate
	Sample size ≥ 100	Hu et al.[4], 2012	HCV-related diseases	3	OR	1.01[0.23 to 4.41]	0.99	Equivalent outcomes	87%; 0.0006	Moderate
**Study design**	Prospective studies	Hu et al.[4], 2012	HCV-related diseases	3	OR	0.57[0.10 to 3.29]	0.53	Equivalent outcomes	88%; 0.0002	Moderate

	Retrospective studies	Hu et al.[4], 2012	HCV-related diseases	4	OR	1.07[0.59 to 1.94]	0.82	Equivalent outcomes	0%; 0.56	Moderate
**Donor age**	Lower in LDLT group	Hu et al.[4], 2012	HCV-related diseases	4	OR	0.99[0.34 to 2.86]	0.99	Equivalent outcomes	71%; 0.01	Moderate
**Median year of follow-up period**	2001 or earlier	Hu et al.[4], 2012	HCV-related diseases	3	OR	0.68[0.21 to 2.26]	0.53	Equivalent outcomes	62%; 0.07	Moderate
	After 2001	Hu et al.[4], 2012	HCV-related diseases	4	OR	0.85[0.26 to 2.79]	0.78	Equivalent outcomes	81%; 0.001	Moderate
**Presence of Only HCC patients**	More in LDLT group	Hu et al.[4], 2012	HCV-related diseases	3	OR	1.79[0.86 to 3.75]	0.12	Equivalent outcomes	51%; 0.28	Moderate
	More in DDLT group	Hu et al.[4], 2012	HCV-related diseases	3	OR	0.53[0.18 to 1.56]	0.25	Equivalent outcomes	66%; 0.05	Moderate
**Ratio of LDLT/DDLT**	＞0.5	Hu et al.[4], 2012	HCV-related diseases	3	OR	1.25[0.66 to 2.38]	0.49	Equivalent outcomes	0%; 0.78	Moderate
	＜0.5	Hu et al.[4], 2012	HCV-related diseases	4	OR	0.53[0.14 to 2.09]	0.37	Equivalent outcomes	82%; 0.0007	Moderate
**1-year Only HCC patients recurrence**										
**Sample size**	DDLT < 100	Tang et al.[8],2020	Only Only HCC patients patients	3	**OR**	**0.56[0.35 to 0.91]**	**0.02**	LDLT	35%; 0.21	Critically low
	DDLT ≥ 100	Tang et al.[8],2020	Only HCC patients	5	OR	1.29[0.62 to 2.71]	0.49	Equivalent outcomes	81%; 0.0002	Critically low
**Milan criteria**	Within Milan criteria	Liang et al.[2],2012	Only HCC patients	2	**OR**	**3.83[1.11 to 13.24]**	**0.03**	DDLT	0%; 0.86	Critically low
**Perdominant sample proportion**	LDLT	Liang et al.[2],2012	Only HCC patients	2	OR	6.99[0.87 to 55.92]	0.07	Equivalent outcomes	0%; 0.44	Critically low

	DDLT	Liang et al.[2],2012	Only HCC patients	2	OR	0.76[0.17 to 3.45]	0.73	Equivalent outcomes	29%; 0.24	Critically low
**3-year Only HCC patients recurrence**										
**Sample size**	DDLT < 100	Tang et al.[8],2020	Only HCC patients	2	OR	0.73[0.46 to 1.14]	0.17	Equivalent outcomes	0%; 0.63	Critically low
	DDLT ≥ 100	Tang et al.[8],2020	Only HCC patients	3	OR	0.99[0.44 to 2.23]	0.98	Equivalent outcomes	89%; 0.0002	Critically low
**Milan criteria**	Within Milan criteria	Liang et al.[2],2012	Only HCC patients	3	OR	4.19[0.57 to 30.82]	0.16	Equivalent outcomes	57%; 0.10	Critically low
	Beyond Milan criteria	Liang et al.[2],2012	Only HCC patients	2	OR	1.77[0.05 to 66.47]	0.76	Equivalent outcomes	79%; 0.03	Critically low
**Perdominant sample proportion**	LDLT	Liang et al.[2],2012	Only HCC patients	2	**OR**	**20.20[2.66 to 153.38]**	**0.004**	DDLT	0%; 0.72	Critically low
	DDLT	Liang et al.[2],2012	Only HCC patients	2	OR	0.83[0.40 to 1.72]	0.62	Equivalent outcomes	0%; 0.45	Critically low
**5-year Only HCC patients recurrence**										
	DDLT < 100	Tang et al.[8],2020	Only HCC patients	4	**OR**	**0.70[0.50 to 0.98]**	**0.04**	LDLT	0%; 0.56	Critically low
	DDLT ≥ 100	Tang et al.[8],2020	Only HCC patients	4	OR	1.19[0.49 to 2.90]	0.7	Equivalent outcomes	91%; < 0.00001	Critically low
**Milan criteria**	Within Milan criteria	Liang et al.[2],2012	Only HCC patients	2	OR	2.55[0.14 to 45.41]	0.52	Equivalent outcomes	72%; 0.06	Critically low
**Perdominant sample proportion**	DDLT	Liang et al.[2],2012	Only HCC patients	2	OR	0.91[0.47 to 1.73]	0.76	Equivalent outcomes	0%; 0.94	Critically low

MA, meta-analysis; CI, confidence interval; RR, relative risk; OR, odds ratio; HR, hazard ratio; NR, not reported; LDLT, living donor liver transplantation; DDLT, deceased donor liver transplantation.

Supplementary Table S5. Characteristics and quality assessment of the meta-analyses comparing the survival of graft between LDLT and DDLT. Significant associations (P<0.05) are presented in bold. Associations reported in italic are those retained in the main analysis.

Outcomes	Author, year	Type of Subjects	No. of studies (T/R/C/P)	No. of LDLT/DDLT	MA metric	Effects model	Risk estimate(95%CI)	P-value	Favours	I^2; Q test P value	Egger test P value	AMSTAR 2 final rating
1-year graft survival	Cavalcante et al.[7],2022	NR	9/0/9/0	NR/NR	**HR**	**fixed**	**1.36[1.16 to 1.60]**	**0.0001**	LDLT	0%;0.90	PB	Critically low
1-year Graft Survival	*Barbetta et al.[6],2021*	*NR*	*7/0/7/0*	*3186/58781*	*HR*	*fixed*	*0.94[0.87 to 1.02]*	*0.14*	*Equivalent outcomes*	*59%;0.02*	*NR*	*Critically low*
1-year Graft Survival	*Hu et al.[4], 2012*	*HCV-related diseases*	*8/0/8/0*	*225/917*	***OR***	***random***	***0.61[0.39 to 0.96]***	***0.03***	*DDLT*	*3%;0.40*	*NPB*	*Moderate*
2-year Graft Survival	*Hu et al.[4], 2012*	*HCV-related diseases*	*6/0/6/0*	*127/509*	*OR*	*random*	*0.61[0.37 to 1.02]*	*0.06*	*Equivalent outcomes*	*0%;0.56*	*NPB*	*Moderate*
3-year graft survival	Cavalcante et al.[7],2022	NR	9/0/9/0	NR/NR	HR	fixed	1.13[0.96 to 1.33]	0.13	Equivalent outcomes	34%;0.15	PB	Critically low

3-year Graft Survival	*Barbetta et al.[6],2021*	*NR*	*8/0/8/0*	*3263/59025*	*HR*	*fixed*	*0.96[0.89 to 1.03]*	*0.25*	*Equivalent outcomes*	*53%;0.04*	*NR*	*Critically low*
3-year Graft Survival	*Hu et al.[4], 2012*	*HCV-related diseases*	*7/0/7/0*	*349/841*	***OR***	***random***	***0.66[0.48 to 0.92]***	***0.01***	*DDLT*	*0%;0.55*	*NPB*	*Moderate*
4-year Graft Survival	*Hu et al.[4], 2012*	*HCV-related diseases*	*3/0/3/0*	*78/397*	*OR*	*random*	*0.74[0.31 to 1.78]*	*0.51*	*Equivalent outcomes*	*56%;0.10*	*NPB*	*Moderate*
5-year graft survival	Cavalcante et al.[7],2022s	NR	7/0/7/0	NR/NR	HR	random	0.99[0.74 to 1.33]	0.96	Equivalent outcomes	78%;0.0001	PB	Critically low
5-year Graft Survival	*Barbetta et al.[6],2021*	*NR*	*6/0/6/0*	*3062/58625*	*HR*	*fixed*	*0.95[0.88 to 1.01]*	*0.12*	*Equivalent outcomes*	*63%;0.02*	*NR*	*Critically low*
5-year Graft Survival	*Hu et al.[4], 2012*	*HCV-related diseases*	*4/0/4/0*	*153/752*	*OR*	*random*	*0.85[0.42 to 1.73]*	*0.66*	*Equivalent outcomes*	*68%;0.03*	*NPB*	*Moderate*
Graft loss due to HCV recurrence	*Hu et al.[4], 2012*	*HCV-related diseases*	*3/0/3/0*	*337/4065*	*OR*	*random*	*1.15[0.49 to 2.68]*	*0.75*	*Equivalent outcomes*	*23%;0.27*	*NPB*	*Moderate*

MA, meta-analysis; CI, confidence interval; T, total No. of studies; R, randomized controlled trial; C, cohort studies; P, population-based case-control and/or cross-sectional studies; RR, relative risk; OR, odds ratio; HR, hazard ratio; NR, not reported; LDLT, living donor liver transplantation; DDLT, deceased donor liver transplantation; NPB, no evidence of publication bias; PB, publication bias.

Supplementary Table S6. Available results of the subgroup meta-analyses comparing the survival of graft between LDLT and DDLT in recipient patients. Significant associations (P<0.05) are presented in bold.

Subgroup analysis	Classification	Author, year	Diagnosis of the patients	No. of studies	MA metric	Risk estimate[95%CI]	P-value	Favours	I^2; Q test P value	AMSTAR final rating
**1-year graft survival**										
**recipients' age**	<50 year	Cavalcante et al.[7],2022	NR	3	**HR**	**1.31[1.08 to 1.59]**	**0.007**	LDLT	0%; NR	Critically low
	≥50 year	Cavalcante et al.[7],2022	NR	3	HR	1.15[0.61 to 2.18]	0.66	Equivalent outcomes	0%; NR	Critically low
**study design**	Prospective studies	Hu et al.[4], 2012	HCV-related diseases	3	**OR**	**0.46[0.24 to 0.87]**	**0.02**	DDLT	0%; 0.75	Moderate
	Retrospective studies	Hu et al.[4], 2012	HCV-related diseases	5	OR	0.81[0.40 to 1.62]	0.55	Equivalent outcomes	25%; 0.25	Moderate
**sample size**	sample size ＜100	Hu et al.[4], 2012	HCV-related diseases	4	OR	0.66[0.22 to 1.95]	0.45	Equivalent outcomes	0%; 0.58	Moderate
	sample size ≥ 100	Hu et al.[4], 2012	HCV-related diseases	4	OR	0.64[0.33 to 1.23]	0.45	Equivalent outcomes	0%; 0.58	Moderate
**median year of follow-up period**	2001 or earlier	Hu et al.[4], 2012	HCV-related diseases	3	OR	0.52[0.25 to 1.05]	0.07	Equivalent outcomes	0%; 0.43	Moderate
	after 2001	Hu et al.[4], 2012	HCV-related diseases	5	OR	0.71[0.34 to 1.46]	0.35	Equivalent outcomes	23%; 0.27	Moderate

**Presence of HCC**	more in LDLT group	Hu et al.[4], 2012	HCV-related diseases	3	**OR**	**0.42[0.19 to 0.94]**	**0.03**	DDLT	0%; 0.55	Moderate
	more in DDLT group	Hu et al.[4], 2012	HCV-related diseases	4	OR	0.82[0.43 to 1.55]	0.53	Equivalent outcomes	22%; 0.28	Moderate
**Ratio of LDLT/DDLT**	＞0.5	Hu et al.[4], 2012	HCV-related diseases	3	OR	0.75[0.28 to 2.00]	0.56	Equivalent outcomes	0%; 0.76	Moderate
	＜0.5	Hu et al.[4], 2012	HCV-related diseases	5	OR	0.61[0.31 to 1.22]	0.16	Equivalent outcomes	39%; 0.16	Moderate
**2-year graft survival**										
**study design**	Prospective studies	Hu et al.[4], 2012	HCV-related diseases	2	OR	0.41[0.12 to 1.36]	0.15	Equivalent outcomes	0%; 0.49	Moderate
	Retrospective studies	Hu et al.[4], 2012	HCV-related diseases	4	OR	0.67[0.38 to 1.18]	0.17	Equivalent outcomes	0%; 0.40	Moderate
**sample size**	sample size ＜100	Hu et al.[4], 2012	HCV-related diseases	4	OR	0.59[0.27 to 1.27]	0.18	Equivalent outcomes	0%; 0.60	Moderate
	sample size ≥ 100	Hu et al.[4], 2012	HCV-related diseases	2	OR	0.65[0.24 to 1.74]	0.39	Equivalent outcomes	52%; 0.15	Moderate
**median year of follow-up period**	2001 or earlier	Hu et al.[4], 2012	HCV-related diseases	3	OR	0.74[0.35 to 1.54]	0.42	Equivalent outcomes	27%; 0.26	Moderate
	after 2001	Hu et al.[4], 2012	HCV-related diseases	3	OR	0.45[0.18 to 1.10]	0.08	Equivalent outcomes	0%; 0.77	Moderate
**Presence of HCC**	more in LDLT group	Hu et al.[4], 2012	HCV-related diseases	3	OR	0.57[0.28 to 1.17]	0.12	Equivalent outcomes	0%; 0.44	Moderate
	more in DDLT group	Hu et al.[4], 2012	HCV-related diseases	2	OR	0.83[0.37 to 1.88]	0.66	Equivalent outcomes	0%; 0.37	Moderate

**Ratio of LDLT/DDLT**	＞0.5	Hu et al.[4], 2012	HCV-related diseases	3	OR	1.04[0.49 to 2.22]	0.92	Equivalent outcomes	0%; 0.87	Moderate
	＜0.5	Hu et al.[4], 2012	HCV-related diseases	3	**OR**	**0.40[0.20 to 0.79]**	**0.009**	DDLT	0%; 0.87	Moderate
**3-year Graft Survival**										
**recipients' age**	<50 year	Cavalcante et al.[7],2022	NR	3	HR	1.04[0.79 to 1.36]	0.8	Equivalent outcomes	64%; NR	Critically low
	≥50 year	Cavalcante et al.[7],2022	NR	3	HR	1.13[0.68 to 1.86]	0.64	Equivalent outcomes	5%; NR	Critically low
**study design**	Prospective studies	Hu et al.[4], 2012	HCV-related diseases	3	**OR**	**0.63[0.42 to 0.96]**	**0.03**	DDLT	0%; 0.75	Moderate
	Retrospective studies	Hu et al.[4], 2012	HCV-related diseases	4	OR	0.74[0.38 to 1.43]	0.37	Equivalent outcomes	30%; 0.24	Moderate
**sample size**	sample size ＜100	Hu et al.[4], 2012	HCV-related diseases	3	OR	0.83[0.39 to 1.79]	0.63	Equivalent outcomes	0%; 0.80	Moderate
	sample size ≥ 100	Hu et al.[4], 2012	HCV-related diseases	4	**OR**	**0.64[0.41 to 0.98]**	**0.04**	DDLT	26%; 0.25	Moderate
**median year of follow-up period**	2001 or earlier	Hu et al.[4], 2012	HCV-related diseases	4	OR	0.66[0.38 to 1.17]	0.15	Equivalent outcomes	37%; 0.19	Moderate
	after 2001	Hu et al.[4], 2012	HCV-related diseases	3	OR	0.72[0.43 to 1.23]	0.23	Equivalent outcomes	0%; 0.99	Moderate
**Presence of HCC**	more in LDLT group	Hu et al.[4], 2012	HCV-related diseases	2	OR	0.59[0.18 to 1.91]	0.38	Equivalent outcomes	55%; 0.14	Moderate

	more in DDLT group	Hu et al.[4], 2012	HCV-related diseases	3	OR	0.84[0.51 to 1.39]	0.5	Equivalent outcomes	0%; 0.60	Moderate
**Ratio of LDLT/DDLT**	＞0.5	Hu et al.[4], 2012	HCV-related diseases	3	OR	0.80[0.43 to 1.49]	0.48	Equivalent outcomes	30%; 0.24	Moderate
	＜0.5	Hu et al.[4], 2012	HCV-related diseases	4	**OR**	**0.60[0.38 to 0.96]**	**0.03**	DDLT	0%; 0.62	Moderate
**4-year Graft Survival**										
**study design**	Retrospective studies	Hu et al.[4], 2012	HCV-related diseases	2	OR	0.79[0.19 to 3.18]	0.74	Equivalent outcomes	78%; 0.03	Moderate
**sample size**	sample size ≥ 100	Hu et al.[4], 2012	HCV-related diseases	2	OR	0.79[0.19 to 3.18]	0.74	Equivalent outcomes	78%; 0.03	Moderate
**median year of follow-up period**	2001 or earlier	Hu et al.[4], 2012	HCV-related diseases	2	OR	0.79[0.19 to 3.18]	0.74	Equivalent outcomes	78%; 0.03	Moderate
**Ratio of LDLT/DDLT**	＜0.5	Hu et al.[4], 2012	HCV-related diseases	2	**OR**	**0.48[0.23 to 0.97]**	**0.04**	DDLT	0%; 0.48	Moderate
**5-year Graft Survival**										
**recipients' age**	<50 year	Cavalcante et al.[7],2022	NR	3	HR	0.89[0.58 to 1.35]	0.57	Equivalent outcomes	87%; NR	Critically low
	≥50 year	Cavalcante et al.[7],2022	NR	2	HR	0.81[0.45 to 1.46]	0.49	Equivalent outcomes	74%; NR	Critically low
**study design**	Retrospective studies	Hu et al.[4], 2012	HCV-related diseases	3	OR	0.89[0.30 to 2.60]	0.82	Equivalent outcomes	78%; 0.01	Moderate

**sample size**	sample size ≥ 100	Hu et al.[4], 2012	HCV-related diseases	4	OR	0.85[0.42 to 1.73]	0.66	Equivalent outcomes	68%; 0.03	Moderate
**median year of follow-up period**	2001 or earlier	Hu et al.[4], 2012	HCV-related diseases	2	OR	0.78[0.11 to 5.46]	0.81	Equivalent outcomes	88%; 0.003	Moderate
	after 2001	Hu et al.[4], 2012	HCV-related diseases	2	OR	0.89[0.55 to 1.45]	0.65	Equivalent outcomes	0%; 0.65	Moderate
**Presence of HCC**	more in DDLT group	Hu et al.[4], 2012	HCV-related diseases	3	OR	1.12[0.65 to 1.93]	0.69	Equivalent outcomes	35%; 0.22	Moderate
**Ratio of LDLT/DDLT**	＜0.5	Hu et al.[4], 2012	HCV-related diseases	3	OR	0.66[0.33 to 1.32]	0.24	Equivalent outcomes	59%; 0.09	Moderate
**Graft loss due to HCV recurrence**										
**study design**	Retrospective studies	Hu et al.[4], 2012	HCV-related diseases	2	OR	1.10[0.12 to 9.91]	0.93	Equivalent outcomes	57%; 0.13	Moderate
**sample size**	sample size ≥ 100	Hu et al.[4], 2012	HCV-related diseases	2	OR	0.96[0.37 to 2.49]	0.93	Equivalent outcomes	35%; 0.22	Moderate
**median year of follow-up period**	2001 or earlier	Hu et al.[4], 2012	HCV-related diseases	3	OR	1.15[0.49 to 2.68]	0.75	Equivalent outcomes	23%; 0.27	Moderate
**Ratio of LDLT/DDLT**	＞0.5	Hu et al.[4], 2012	HCV-related diseases	3	OR	1.15[0.49 to 2.68]	0.75	Equivalent outcomes	23%; 0.27	Moderate

MA, meta-analysis; CI, confidence interval; RR, relative risk; OR, odds ratio; HR, hazard ratio; NR, not reported; LDLT, living donor liver transplantation; DDLT, deceased donor liver transplantation.

Supplementary Table S7. Characteristics and quality assessment of the meta-analyses comparing the perioperative outcomes between LDLT and DDLT. Significant associations (P<0.05) are presented in bold. Associations reported in italic are those retained in the main analysis.

Outcomes	Author, year	Type of Subjects	No. of studies (T/R/C/P)	No. of LDLT/DDLT	MA metric	Effects model	Risk estimate(95%CI)	P-value	Favours	I^2; Q test P value	Egger test P value	AMSTAR 2 final rating
Time on Waiting List	*Barbetta et al.[6],2021*	*NR*	*8/0/8/0*	*3237/58900*	***MD***	***random***	***-71.43[-101.42 to -41.44]***	***<0.00001***	*LDLT*	*97%; < 0.00001*	*NR*	*Critically low*
MELD score at transplant	*Barbetta et al.[6],2021*	*NR*	*14/0/14/0*	*3776/59399*	***MD***	***random***	***-2.54[-5.02 to -0.06]***	***0.04***	*LDLT*	*98%; < 0.00001*	*NR*	*Critically low*
CIT	Tang et al.[8],2020	NR	6/0/6/0	1608/3234	**WMD**	**random**	**-373.39[-399.41 to -347.37]**	**<0.00001**	LDLT	96%; < 0.00001	PB	Critically low
CIT	*Wan et al.[10],2014*	*NR*	*7/0/7/0*	*886/724*	***MD***	***random***	***-346.28[-417.1 to -275.46]***	***<0.00001***	*LDLT*	*99%; < 0.00001*	*NPB*	*Low*
Allogeneic RBC Transfusion	Tang et al.[8],2020	NR	4/0/4/0	294/1029	WMD	fixed	0.69[-0.14 to 1.51]	0.1	Equivalent outcomes	0%;0.71	NPB	Critically low
Allogeneic RBC Transfusion	*Wan et al.[10],2014*	*NR*	*5/0/5/0*	*354/524*	*MD*	*random*	*-0.85[-3.37 to 1.66]*	*0.51*	*Equivalent outcomes*	*72%;0.006*	*NPB*	*Low*
DRO	Tang et al.[8],2020	NR	4/0/4/0	858/6844	**WMD**	**fixed**	**141.68[129.19 to 154.16]**	**<0.00001**	DDLT	47%;0.13	NPB	Critically low
DRO	*Wan et al.[10],2014*	*NR*	*5/0/5/0*	*634/555*	***MD***	***random***	***2.80[2.18 to 3.42]***	***<0.00001***	*DDLT*	*76%;0.003*	*NPB*	*Low*
Length of Hospital Stay	Tang et al.[8],2020	NR	4/0/4/0	611/6861	WMD	fixed	1.82[-0.91 to 4.56]	0.19	Equivalent outcomes	0%;0.61	NPB	Critically low

Length of Hospital Stay	Barbetta et al.[6],2021	NR	8/0/8/0	3640/65065	MD	random	-3.80[-8.36 to 0.76]	0.1	Equivalent outcomes	95%; < 0.00001	NR	Critically low
Length of Hospital Stay	*Wan et al.[10],2014*	*NR*	*4/0/4/0*	*606/605*	*MD*	*fixed*	*-1.37[-4.37 to 1.64]*	*0.37*	*Equivalent outcomes*	*48%;0.12*	*NPB*	*Low*
Perioperative mortality within 3 months	*Zhu et al.[1],2019*	*Only HCC patients*	*7/0/7/0*	*474/1017*	*RR*	*fixed*	*0.89[0.50 to 1.59]*	*0.7*	*Equivalent outcomes*	*34%;0.17*	*0.687*	*Critically low*
perioperative mortality rates	Tang et al.[8],2020	NR	10/0/10/0	2174/16547	OR	fixed	1.03[0.81 to 1.29]	0.82	Equivalent outcomes	39%;0.10	NPB	Critically low
Perioperative mortality rates	*Wan et al.[10],2014*	*NR*	*4/0/4/0*	*790/843*	*OR*	*fixed*	*1.23[0.80 to 1.89]*	*0.34*	*Equivalent outcomes*	*49%;0.12*	*NPB*	*Low*

MA, meta-analysis; CI, confidence interval; T, total No. of studies; R, randomized controlled trial; C, cohort studies; P, population-based case-control and/or cross-sectional studies; MD, mean difference; WMD, weighted mean difference; RR, relative risk; OR, odds ratio; HR, hazard ratio; NR, not reported; LDLT, living donor liver transplantation; DDLT, deceased donor liver transplantation; NPB, no evidence of publication bias; PB, publication bias; CIT, cold ischemia time; RBC, red blood cell; DRO, duration of the recipient operation;

Supplementary Table S8. Available results of the subgroup meta-analyses comparing the perioperative outcomes between LDLT and DDLT in recipient patients. Significant associations (P<0.05) are presented in bold.

Subgroup analysis	Classification	Author, year	Diagnosis of the patients	No. of studies	MA metric	Risk estimate[95%CI]	P-value	Favours	I^2; Q test P value	AMSTAR final rating
**CIT**										
**Sample size**	DDLT < 400	Tang et al.[8],2020	NR	3	**MD**	**-371.10[-373.60 to -368.60]**	**< 0.00001**	LDLT	37%; 0.20	Critically low
	DDLT ≥ 400	Tang et al.[8],2020	NR	3	**MD**	**-372.02[-445.71 to -298.34]**	**< 0.00001**	LDLT	97%; < 0.00001	Critically low
**transplant area**	Western	Wan et al.[10],2014	NR	4	**MD**	**-414.03[-465.94 to -362.12]**	**<0.001**	LDLT	87%; ＜ 0.001	Low
	Eastern	Wan et al.[10],2014	NR	3	**MD**	**-235.42[-244.88 to -225.97]**	**<0.001**	LDLT	0%; 0.56	Low
**DRO**		Wan et al.[10],2014								
**study design**	Prospective cohort	Wan et al.[10],2014	NR	2	**MD**	**3.60[2.98 to 4.22]**	**<0.001**	DDLT	0%; ＞0.99	Low
	Retrospective cohort	Wan et al.[10],2014	NR	3	**MD**	**2.32[2.06 to 2.59]**	**<0.001**	DDLT	22%; 0.28	Low
**Allogeneic RBC transfusion requirement**										
**study design**	Prospective cohort	Wan et al.[10],2014	NR	2	MD	-3.54[-11.74 to 4.67]	0.4	Equivalent outcomes	62%; 0.10	Low
	Retrospective cohort	Wan et al.[10],2014	NR	3	MD	0.81[-0.21 to 1.83]	0.12	Equivalent outcomes	0%; 0.53	Low

MA, meta-analysis; CI, confidence interval; MD, mean difference; RR, relative risk; OR, odds ratio; HR, hazard ratio; NR, not reported; LDLT, living donor liver transplantation; DDLT, deceased donor liver transplantation; CIT, cold ischemia time; RBC, red blood cell; DRO, duration of the recipient operation.

Supplementary Table S9. Characteristics and quality assessment of the meta-analyses comparing the postoperative complications and retransplantation rate between LDLT and DDLT. Significant associations (P<0.05) are presented in bold. Associations reported in italic are those retained in the main analysis.

Outcomes	Author, year	Type of Subjects	No. of studies (T/R/C/P)	No. of LDLT/DDLT	MA metric	Effects model	Risk estimate(95%CI)	P-value	Favours	I^2; Q test P value	Egger test P value	AMSTAR 2 final rating
Postoperative Intra-Abdominal Bleeding Rates	Tang et al.[8],2020	NR	6/0/6/0	1137/7179	**OR**	**fixed**	**0.64[0.46 to 0.88]**	**0.006**	LDLT	8%;0.37	NPB	Critically low
Postoperative Intra-Abdominal Bleeding Rates	*Wan et al.[10],2014*	*NR*	*3/0/3/0*	*599/552*	*OR*	*fixed*	*0.72[0.43 to 1.20]*	*0.21*	*Equivalent outcomes*	*32%;0.23*	*NPB*	*Low*
Hepatic Artery Thrombosis	Barbetta et al.[6],2021	NR	7/0/7/0	483/846	OR	random	2.07[0.84 to 5.09]	0.11	Equivalent outcomes	0%;0.99	NR	Critically low
Vascular Complications	Tang et al.[8],2020	NR	6/0/6/0	828/1142	**OR**	**fixed**	**2.00[1.31 to 3.07]**	**0.001**	DDLT	45%;0.11	NPB	Critically low
Vascular Complications	*Wan et al.[10],2014*	*NR*	*4/0/4/0*	*702/659*	***OR***	***fixed***	***2.16[1.32 to 3.54]***	***0.002***	*DDLT*	*0%;0.73*	*NPB*	*Low*
Biliary Complications	Tang et al.[8],2020	NR	14/0/14/0	1916/9362	**OR**	**random**	**2.23[1.59 to 3.13]**	**<0.00001**	DDLT	77%;＜ 0.00001	PB	Critically low
Biliary Complications	Barbetta et al.[6],2021	NR	11/0/11/0	1159/7489	**OR**	**random**	**2.14[1.76 to 2.59]**	**＜0.00001**	DDLT	0%;0.60	NR	Critically low
Biliary Complications	*Wan et al.[10],2014*	*NR*	*8/0/8/0*	*687/1819*	***OR***	***random***	***3.08[1.97 to 4.81]***	***＜0.00001***	*DDLT*	*63%;0.008*	*NPB*	*Low*
Risk of Infection	*Barbetta et al.[6],2021*	*NR*	*8/0/8/0*	*859/7084*	*OR*	*random*	*0.67[0.42 to 1.09]*	*0.11*	*Equivalent outcomes*	*75%;0.0002*	*NR*	*Critically low*

Acute rejection episodes	*Hu et al.[4], 2012*	*HCV-related diseases*	*11/0/11/0*	*379/873*	*OR*	*random*	*0.83[0.53 to 1.30]*	*0.41*	*Equivalent outcomes*	*37%;0.10*	*0.032*	*Moderate*
Rejection	*Barbetta et al.[6],2021*	*NR*	*11/0/11/0*	*1292/7881*	***OR***	***random***	***0.72[0.55 to 0.95]***	***0.02***	*LDLT*	*0%;0.58*	*NR*	*Critically low*
Complications	Zhu et al.[1],2019	*Only HCC patients*	*2/0/2/0*	*78/147*	*RR*	*fixed*	*0.99[0.70 to 1.39]*	*0.94*	*Equivalent outcomes*	*0%;0.34*	*0.687*	*Critically low*
Retransplantation Rate	Tang et al.[8],2020	NR	8/0/8/0	725/2257	OR	fixed	1.29[0.87 to 1.93]	0.21	Equivalent outcomes	29%;0.20	NPB	Critically low
Retransplantation Rate	*Wan et al.[10],2014*	*NR*	*7/0/7/0*	*455/1440*	***OR***	***fixed***	***1.76[1.09 to 2.83]***	***0.02***	*DDLT*	*25%;0.24*	*NPB*	*Low*

MA, meta-analysis; CI, confidence interval; T, total No. of studies; R, randomized controlled trial; C, cohort studies; P, population-based case-control and/or cross-sectional studies; RR, relative risk; OR, odds ratio; NR, not reported; LDLT, living donor liver transplantation; DDLT, deceased donor liver transplantation; NPB, no evidence of publication bias; PB, publication bias.

Supplementary Table S10. Available results of the subgroup meta-analyses comparing the postoperative complications and retransplantation rate between LDLT and DDLT in recipient patients. Significant associations (P<0.05) are presented in bold.

Subgroup analysis	Classification	Author, year	Diagnosis of the patients	No. of studies	MA metric	Risk estimate[95%CI]	P-value	Favours	I^2; Q test P value	AMSTAR final rating
**Acute rejection**										

**study design**	Prospective studies	Hu et al.[4], 2012	HCV-related diseases	5	OR	1.36[0.91 to 2.05]	0.14	Equivalent outcomes	0%; 0.67	Moderate
	Retrospective studies	Hu et al.[4], 2012	HCV-related diseases	6	OR	0.58[0.32 to 1.04]	0.07	Equivalent outcomes	28%; 0.23	Moderate
**sample size**	sample size ＜100	Hu et al.[4], 2012	HCV-related diseases	7	OR	0.71[0.41 to 1.25]	0.24	Equivalent outcomes	0%; 0.82	Moderate
	sample size ≥ 100	Hu et al.[4], 2012	HCV-related diseases	4	OR	0.90[0.40 to 2.04]	0.8	Equivalent outcomes	74%; 0.009	Moderate
**Donor age**	lower in LDLT group			3	OR	0.55[0.11 to 2.76]	0.47	Equivalent outcomes	75%; 0.02	Moderate
	equivalent			4	OR	1.28[0.85 to 1.93]	0.23	Equivalent outcomes	0%; 0.58	Moderate
**median year of follow-up period**	2001 or earlier	Hu et al.[4], 2012	HCV-related diseases	5	OR	1.17[0.80 to 1.73]	0.42	Equivalent outcomes	0%; 0.54	Moderate
	after 2001	Hu et al.[4], 2012	HCV-related diseases	6	OR	0.66[0.30 to 1.44]	0.29	Equivalent outcomes	50%; 0.07	Moderate
**Presence of HCC**	more in LDLT group	Hu et al.[4], 2012	HCV-related diseases	6	OR	0.98[0.61 to 1.58]	0.94	Equivalent outcomes	50%; 0.53	Moderate
	more in DDLT group	Hu et al.[4], 2012	HCV-related diseases	2	OR	0.36[0.09 to 1.41]	0.14	Equivalent outcomes	49%; 0.16	Moderate
**Ratio of LDLT/DDLT**	＞0.5	Hu et al.[4], 2012	HCV-related diseases	4	OR	1.26[0.83 to 1.91]	0.28	Equivalent outcomes	0%; 0.45	Moderate
	＜0.5	Hu et al.[4], 2012	HCV-related diseases	7	OR	0.64[0.34 to 1.22]	0.18	Equivalent outcomes	39%; 0.13	Moderate
**Vascular complication rates**										
**sample size**	LDLT < 100	Tang et al.[8],2020	NR	3	OR	1.05[0.51 to 2.16]	0.89	Equivalent outcomes	30%; 0.24	Critically low
	LDLT ≥ 100	Tang et al.[8],2020	NR	3	**OR**	**2.89[1.62 to 5.16]**	**0.0003**	**DDLT**	26%; 0.26	Critically low

**Biliary complication rates**										
**sample size**	LDLT < 100	Tang et al.[8],2020	NR	6	**OR**	**3.71[1.58 to 8.71]**	**0.003**	**DDLT**	82%; < 0.0001	Critically low
	LDLT ≥ 100	Tang et al.[8],2020	NR	8	**OR**	**1.77[1.27 to 2.48]**	**0.0008**	**DDLT**	72%; 0.0008	Critically low
	＜50LDLT	Wan et al.[10],2014	NR	3	**OR**	**7.66[4.23 to 13.85]**	**＜0.001**	**DDLT**	0%; 0.38	Low
	≥50LDLT	Wan et al.[10],2014	NR	5	**OR**	**2.11[1.61 to 2.77]**	**＜0.001**	**DDLT**	8%; 0.36	Low
	DDLT < 100	Tang et al.[8],2020	NR	5	**OR**	**4.80[2.88 to 7.98]**	**< 0.00001**	**DDLT**	49%; 0.10	Critically low
	DDLT ≥ 100	Tang et al.[8],2020	NR	9	**OR**	**1.74[1.24 to 2.43]**	**0.001**	**DDLT**	77%; < 0.0001	Critically low
**Diagnosis of the patients**	HCC related	Tang et al.[8],2020	NR	2	OR	2.21[1.72 to 2.83]	< 0.00001	DDLT	50%; 0.16	Critically low
	Not HCC related	Tang et al.[8],2020	NR	12	OR	2.21[1.46 to 3.34]	0.0002	DDLT	79%; < 0.00001	Critically low

MA, meta-analysis; CI, confidence interval; RR, relative risk; OR, odds ratio; NR, not reported; LDLT, living donor liver transplantation; DDLT, deceased donor liver transplantation.

Reference

1. Zhu B, Wang J, Li H, Chen X, Zeng Y. Living or deceased organ donors in liver transplantation for hepatocellular carcinoma: a systematic review and meta-analysis. *HPB : the official journal of the International Hepato Pancreato Biliary Association.* 2019;21(2):133-147.
2. Liang W, Wu L, Ling X, et al. Living donor liver transplantation versus deceased donor liver transplantation for hepatocellular carcinoma: a meta-analysis. *Liver transplantation : official publication of the American Association for the Study of Liver Diseases and the International Liver Transplantation Society.* 2012;18(10):1226-1236.
3. Grant RC, Sandhu L, Dixon PR, Greig PD, Grant DR, McGilvray ID. Living vs. deceased donor liver transplantation for hepatocellular carcinoma: a systematic review and meta-analysis. *Clinical transplantation.* 2013;27(1):140-147.
4. Hu A, Liang W, Zheng Z, Guo Z, He X. Living donor vs. deceased donor liver transplantation for patients with hepatitis C virus-related diseases. *Journal of hepatology.* 2012;57(6):1228-1243.
5. Elkomos BE, Abdo M, Mamdouh R, Abdelaal A. Can living donor liver transplantation provide similar outcomes to deceased-donor liver transplantation for hepatocellular carcinoma? A systematic review and meta-analysis. *Hepatology international.* 2023;17(1):18-37.
6. Barbetta A, Aljehani M, Kim M, et al. Meta-analysis and meta-regression of outcomes for adult living donor liver transplantation versus deceased donor liver transplantation. *American journal of transplantation : official journal of the American Society of Transplantation and the American Society of Transplant Surgeons.* 2021;21(7):2399-2412.
7. Cavalcante LN, Queiroz RMT, Paz C, Lyra AC. BETTER LIVING DONOR LIVER TRANSPLANTATION PATIENT SURVIVAL COMPARED TO DECEASED DONOR - A SYSTEMATIC REVIEW AND META-ANALYSIS. *Arquivos de gastroenterologia.* 2022;59(1):129-136.
8. Tang W, Qiu JG, Cai Y, Cheng L, Du CY. Increased Surgical Complications but Improved Overall Survival with Adult Living Donor Compared to Deceased Donor Liver Transplantation: A Systematic Review and Meta-Analysis. *BioMed research international.* 2020;2020:1320830.
9. Zhang HM, Shi YX, Sun LY, Zhu ZJ. Hepatocellular carcinoma recurrence in living and deceased donor liver transplantation: a systematic review and meta-analysis. *Chinese medical journal.* 2019;132(13):1599-1609.
10. Wan P, Yu X, Xia Q. Operative outcomes of adult living donor liver transplantation and deceased donor liver transplantation: a systematic review and meta-analysis. *Liver transplantation : official publication of the American Association for the Study of Liver Diseases and the International Liver Transplantation Society.* 2014;20(4):425-436.
